# Insights into genomic structure and evolutionary processes of coastal *Suaeda* species in East Asia using cpDNA, nDNA, and genome-wide SNPs

**DOI:** 10.1038/s41598-020-78041-7

**Published:** 2020-12-01

**Authors:** Jong-Soo Park, Dong-Pil Jin, Byoung-Hee Choi

**Affiliations:** Department of Biological Sciences, Inha University, Incheon, 22212 Republic of Korea

**Keywords:** Plant evolution, Phylogenetics, Population genetics, Taxonomy

## Abstract

Species in the genus *Suaeda* have few diagnostic characters and substantial morphological plasticity. Hence, regional floras do not provide clear taxonomic information for *Suaeda* spp. in East Asia. In order to assess the taxonomy of four species in the genus *Suaeda* (*S*. *australis*, *S*. *maritima*, *S*. *japonica*, and *S*. *heteroptera*), cpDNA (*rpl32*-*trnL* and *trnH*-*psbA*), nDNA (ITS), and MIG-seq analyses were carried out. Genome-wide SNP results indicated three lineages: (1) *S*. *australis* in Korea and *S*. *maritima* in Japan, (2) *S*. *maritima* in Korea and *S*. *heteroptera* in China*, *and (3) *S*. *japionica*. In phylogenetic trees and genotype analyses, cpDNA and nDNA results showed discrepancies, while *S*. *japonica* and *S*. *maritima* in Korea, and *S*. *heteroptera* in China shared the same haplotype and ribotype. We suggest that the shared haplotype may be due to chloroplast capture. Based on our results, we assume that *S*. *japonica* was formed by homoploid hybrid speciation between the two lineages.

## Introduction

The genus *Suaeda* Forssk. ex J.F. Gmel., in the Amaranthaceae family^1^, comprises approximately 80–100 species of halophytic herbs or shrubs distributed worldwide in semiarid and arid regions, and along the seashores, including the Pacific^2–5^. An integrated molecular and morphological phylogenetic study of the subfamily Suaedoideae Ulbr. suggests that the genus *Suaeda* is comprised of two subgenera (*Brezia* (Moq.) Freitag & Schütze, and *Suaeda*) and eight sections (*Brezia* (Moq.) Volk. in Engl. & Prantl, *Schanginia* (C.A. Mey.) Volk. in Engl. & Prantl, *Borszczowia* (Bunge) Freitag & Schütze, *Suaeda*, *Physophora* Iljin, *Schoberia* (C.A. Mey.) Volk. in Engl. & Prantl, *Salsina* Moq., and *Helicilla* (Moq.) Baillon in Baillon)^6,7^. There are approximately 20 *Suaeda* species that occur in Korea, China, and Japan, and about six of these inhabit the coastal regions of these countries.

*Suaeda* species are difficult to identify due to a lack of diagnostic characters and substantial morphological plasticity depending on local ecological conditions^6,8^. The descriptions of each species in regional floras and taxonomic studies do not provide consistent information on *S*. *maritima* (L.) Dumort. and related species in East Asia^2, 5, 9–11^. *S*. *maritima*, which according to most authors is distributed in Korea, Japan, and Europe, has an erect and much branched stem, narrowly linear leaves, an acute or subacute apex, and an unchanged perianth when in fruit^5, 9^. However, Hara separated the Japanese populations as subsp. *asiatica* because they differ in their diploid karyotype (2n = 18) from the tetraploid (2n = 36) European plants, and it generally has decumbent or spreading-ascending branches^5, 12, 13^. During our field investigation, we found that the morphological characters of *S*. *maritima* in Japan are similar to those of *S*. *australis* in Korea, including the decumbent or erect stem, and the shapes of the perianth when in fruit^9, 14^. *S*. *australis*, which was described in Australia, was recently reported in Korea, based on its occurrence in southern China, and on morphological characters such as a much branched stem at the base, conspicuous leaf scars, and an unchanged perianth when in fruit^14^. In terms of leaf base articulation, however, *S*. *australis* found in Korea differs from *S*. *australis* described in China^2^. In addition, Lee et al.^15^ indicated that *S*. *australis* found in Korea should be named *S*. *maritima*. *S*. *japonica* Makino is found in mud flats in Korea and Japan, and can be distinguished from other *Suaeda* species found in East Asia by its clavate and obovate or oblanceolate leaves with obtuse apex. In a phylogenetic study on Korean *Suaeda* species, *S*. *japonica*, *S*. *maritima*, and *S*. *australis*, excluding *S*. *malacosperma*, were found to have a discordant relationship among the related species between its ribosomal DNA and chloroplast DNA markers^15, 16^. *S*. *malacosperma* was once treated as a variety of *S*. *maritima*^17^, but is recognized as a different species and a recently studied phylogenetic structure^18^. *S*. *salsa* (L.) Pall. is widely distributed from China to Europe^2^. Lomonosova and Freitag^4^, and Lomonosova et al.^19^ proposed that a lineage of *S*. *salsa* in eastern Eurasia should be considered as *S*. *heteroptera* Kitag. based on differences in morphological traits, karyotypes, and molecular phylogenetic analysis. Lee^20^ recorded *S*. *heteroptera* in Korea with narrower upper leaves than those of *S*. *maritima*. Therefore, it is necessary to confirm the identities of these species, and to determine the relationships between them in order to clarify their taxonomy. In this study, we considered *S*. *salsa* s.l. found in China to be *S*. *heteroptera,* in agreement with Lomonosova et al.^19^. We focused our analyses on *S*. *maritima* and *S*. *australis* in Korea, and *S*. *japonica* and *S*. *heteroptera* in China, as the relationships between these four species are unclear.Previous combined molecular and morphological phylogenetic studies on the genus *Suaeda* by Schütze et al.^6^ have contributed to the clarification of the taxonomic system of the subgenus and section ranks in this genus, while recent molecular phylogenetic studies have found unclear relationships among *Suaeda* spp. at the species level^8, 16^. We used MIG-seq to yield genome-wide SNPs in order to obtain genetic relationship data at the genome and population level. Next-generation sequencing, population genomic approaches, such as restriction site-associated DNA sequencing (RADseq), and multiplexed inter-simple sequence repeat genotyping by sequencing (MIG-seq)^21, 22^, are applicable and convenient methods for this type of analysis^23–25^. We used MIG-seq for this study, which is a PCR-based method for producing several hundred genome-wide single nucleotide polymorphisms (SNPs). MIG-seq has the advantage of being applicable to small quantities of DNA, and MIG-seq has been used to study a diverse number of organisms, including plants^18, 22, 25, 26^. We also included previously used chloroplast and nuclear markers to compare analyses of genome-wide SNP data.

In this study, we aimed to (1) compare the genetic structures of the four targeted coastal *Suaeda* species in East Asia (*S*. *maritima*, *S*. *australis*, *S*. *japonica,* and *S*. *heteroptera*), (2) clarify the taxonomy of these four *Suaeda* species in East Asia by using genetic markers and genome-wide SNPs, and (3) provide insight into the evolution of East Asian *Suaeda* species.

## Results

### Genotype network and distribution

**Figure 1:**
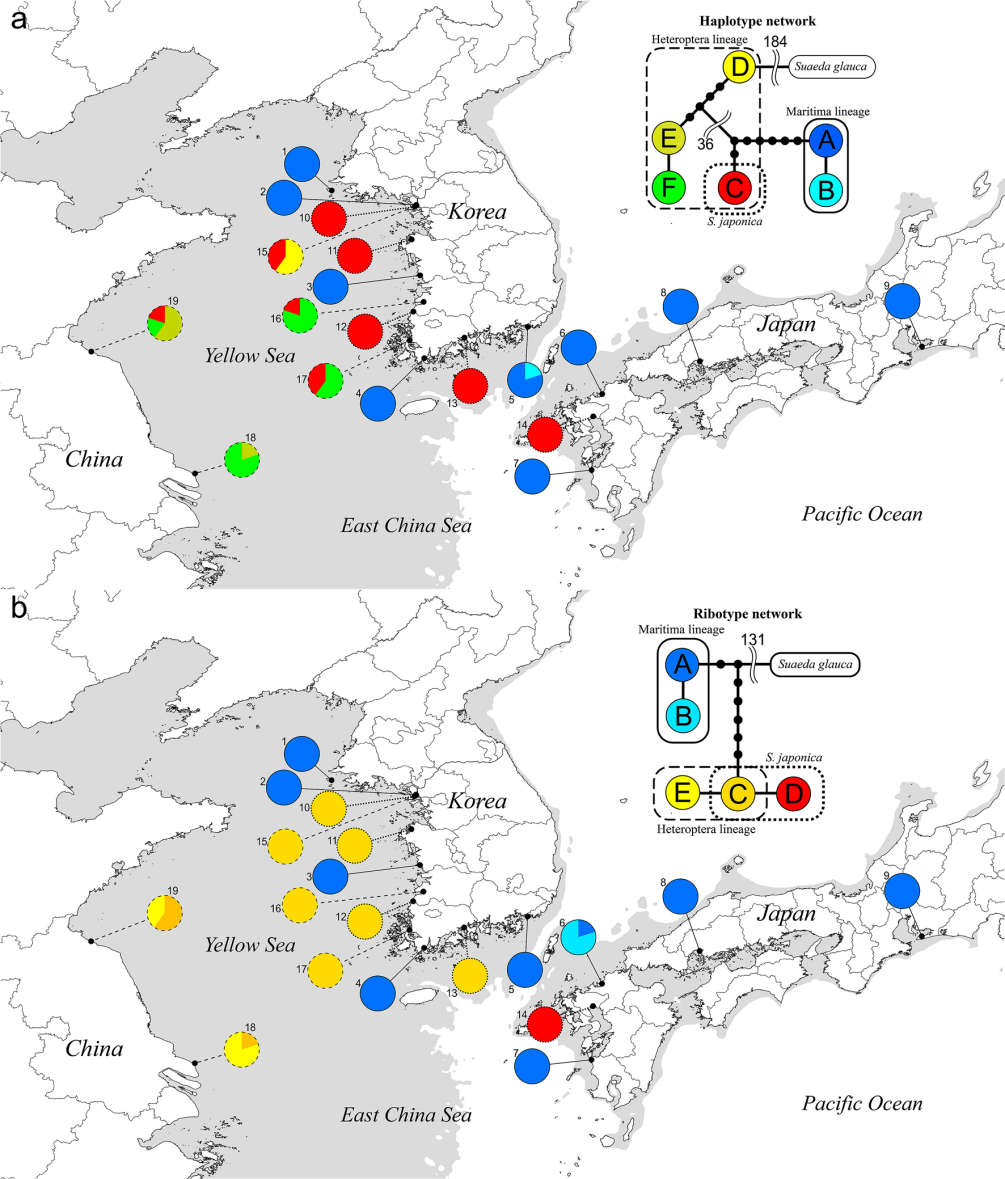
Networks and distributions of genotype in East Asia. (**a**) Haplotype network and distribution. (**b**) Ribotype network and distribution. Solid, dotted, and dashed lines correspond to Maritima lineage, *S*. *japonica* and Heteroptera lineage, respectively. The genotype networks were generated by TCS 1.21^27^ (http://bioresearch.byu.edu/tcs/). Each dot in genotype network indicates mutation step. Background map was created using QGIS 3.6.0 (https://www.qgis.org/), Adobe Illustrator CC 2018 (https://www.adobe.com/), and boundary data of DIVA-GIS (https://www.diva-gis.org/). Grey area indicates dry land in Last Glacial Maximum according to elevation data of Climatologies at High Resolution for the Earth’s Land Surface Areas^28^ (CHELSA, http://chelsa-climate.org/).

Genotypes were identified in five individuals per population using cpDNA and nuclear markers (Fig. 1, Table 1). Six haplotypes were determined from cpDNA sequences concatenated from *rpl32*-*trnL* and *psbA*-*trnH* (Fig. 1a). Haplotype lengths were 1254–1266 bp (excluding *S*. *glauca*, which was used as an outgroup), and the aligned sequence length was 1322 bp. In the haplotype network, three branches were formed from the outgroup *S*. *glauca*. The first branch (haplotypes D, E, and F) was separated from the other branches by at least 40 mutation steps. The second branch (haplotype C) was separated from the third branch (haplotypes A and B) by seven mutation steps.

**Table 1:** Genetic information of samples of *Suaeda australis*, *S*. *maritima*, *S*. *japonica* and *S*. *heteroptera* in Korea, Japan, and China.

Species	Pop no.	N	Hap	Rib	Sites	Var	P	H_O_	H_E_	π	F_IS_
**Maritima lineage**											
*S*. *australis* in Korea	1	8	A(5)	A(5)	16,329	77	2	0.1320	0.0933	0.1007	− 0.0191
	2	10	A(5)	A(5)	11,371	54	1	0.1053	0.0817	0.0867	− 0.0254
	3	11	A(5)	A(5)	11,647	56	4	0.1110	0.1055	0.1111	0.0161
	4	10	A(5)	A(5)	13,245	63	9	0.0789	0.1066	0.1130	0.1158
	5	11	A(4), B(1)	A(5)	10,570	49	2	0.0437	0.0495	0.0523	0.0297
*S*. *maritima* in Japan	6	10	A(5)	A(1), B(4)	15,524	77	5	0.0599	0.0520	0.0552	0.0080
	7	10	A(5)	A(5)	13,112	64	7	0.0430	0.0274	0.0291	− 0.0277
	8	8	A(5)	A(5)	17,129	74	3	0.0405	0.0288	0.0313	− 0.0182
	9	11	A(5)	A(5)	13,391	62	2	0.0392	0.0322	0.0340	− 0.0107
Mean					13,590.9	64.0	3.9	0.0726	0.0641	0.0682	0.0076
*S*. *japonica*	10	11	C(5)	C(5)	7,760	34	4	0.1161	0.1379	0.1457	0.0574
	11	10	C(5)	C(5)	8,433	38	6	0.1310	0.1796	0.1906	0.1546
	12	11	C(5)	C(5)	3,211	17	1	0.0673	0.0846	0.0893	0.0846
	13	11	C(5)	C(5)	7,361	34	3	0.1180	0.1786	0.1887	0.2000
	14	6	C(5)	D(5)	13,527	60	10	0.1272	0.1024	0.1133	− 0.0097
Mean					8,058.4	36.6	4.8	0.1119	0.1366	0.1455	0.0974
**Heteroptera lineage**											
*S*. *maritima* in Korea	15	11	C(2), D(3)	C(5)	5,222	33	3	0.0926	0.1574	0.1660	0.2382
	16	10	C(1), F(4)	C(5)	6,957	34	1	0.0877	0.1551	0.1648	0.2160
	17	11	C(2), F(3)	C(5)	5,354	28	6	0.1340	0.2277	0.2402	0.2817
*S*. *heteroptera*	18	10	E(1), F(4)	C(2), E(3)	7,627	43	1	0.0934	0.1432	0.1524	0.2101
	19	11	C(1), E(3), F(4)	C(3), E(2)	5,087	31	2	0.0601	0.1192	0.1257	0.2389
Mean					6,049.4	33.8	2.6	0.0936	0.1605	0.1698	0.2370

*N* number of samples, *Hap* haplotype, *Rib* ribotype, *Site* nucleotide sites across the data set, *Var* variant nucleotides in each population, *P* number of private alleles, *H*_*O*_ mean observed heterozygosity, *H*_*E*_ mean expected heterozygosity, *π* mean value of nucleotide diversity, *F*_*IS*_ mean value of inbreeding coefficient, *H*_*O*_*, H*_*E*_*, π, and F*_*IS*_ were calculated at variable nucleotide sites.

Haplotypes A and B were identified in *S*. *australis* in Korea, and haplotype A was shared with *S*. *maritima* in Japan. Haplotypes C, D, E, and F were confirmed in *S*. *maritima* in Korea, as were haplotypes C, E*,* and F in *S*. *heteroptera* in China. Haplotype A was found in all *S*. *australis* from Korea and *S*. *maritima* in Japan (Maritima lineage). Haplotype C was found in most populations of *S*. *maritima* in Korea, *S*. *heteroptera* in China (Heteroptera lineage), and *S*. *japonica*, except for population 18.

Ribotypes were 672 bp in length, and the aligned sequence length was 684 bp, including the outgroup. Five ribotypes were identified, and two branches were formed in the ribotype network (Fig. 1b). One branch consisted of ribotypes A and B, and the other consisted of three ribotypes centered around ribotype C. Ribotypes A and B were identified in the Maritima lineage, and ribotype B was only distributed in six populations in Japan (Fig. 1b, Table 1). Ribotypes C and D were found in *S*. *japonica*, and ribotype D was only found in population 14 in Japan. Ribotypes C and E were found in the Heteroptera lineage, and ribotype E ribotype was limited to two populations in China.

### Population genetic analyses based on genome-wide SNPs data

**Figure 2:**
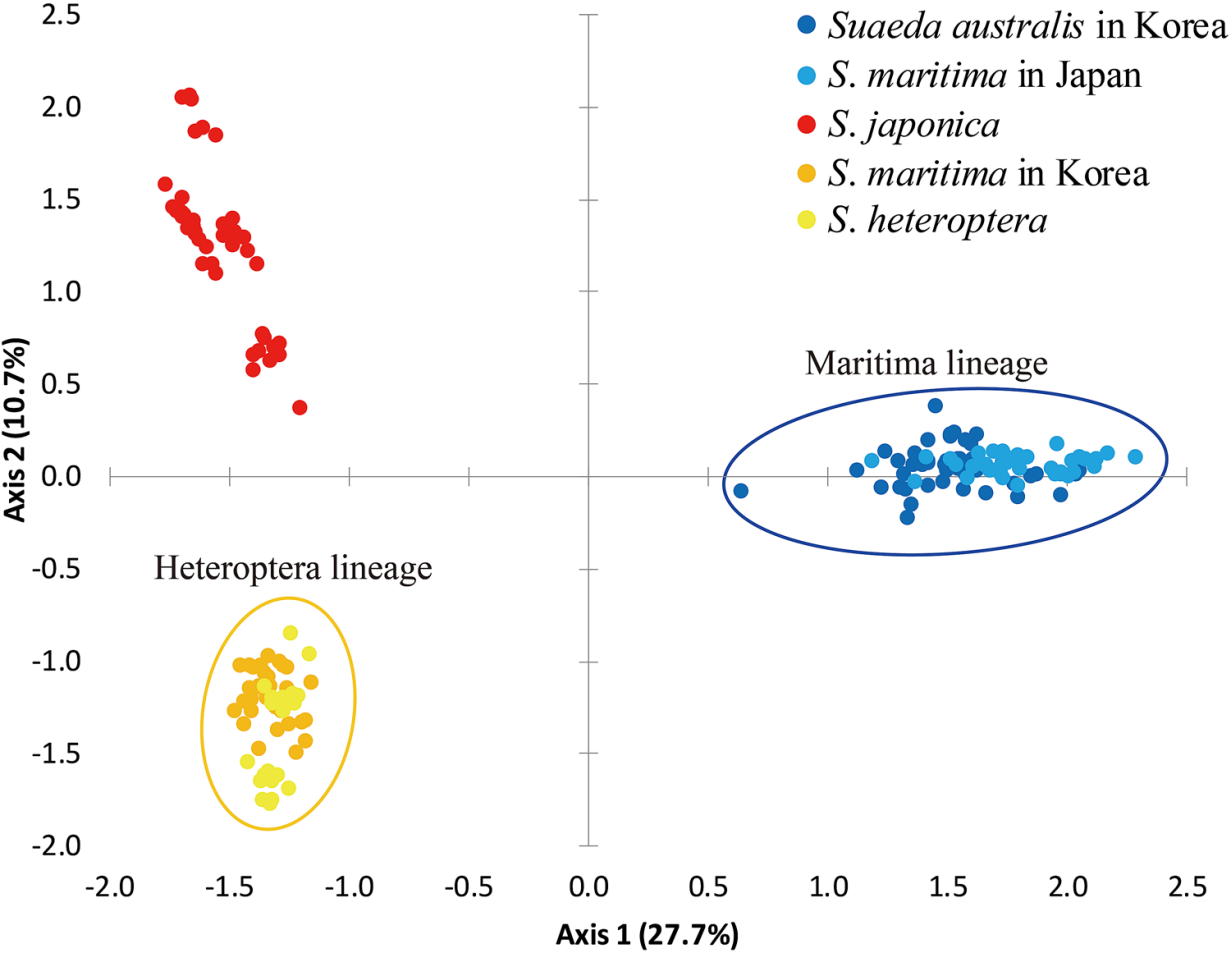
PCoA with genome-wide SNPs by using GenAlEx 6.51b2^29,30^ (https://biology-assets.anu.edu.au/GenAlEx/). The scatter plot was modified with Adobe Illustrator CC 2018 (https://www.adobe.com/). Each dot indicates individual. The studied species are marked by the same colours as in Fig. 1. Maritima and Heteroptera lineages were grouped by blue and orange circles, respectively.

**Figure 3:**
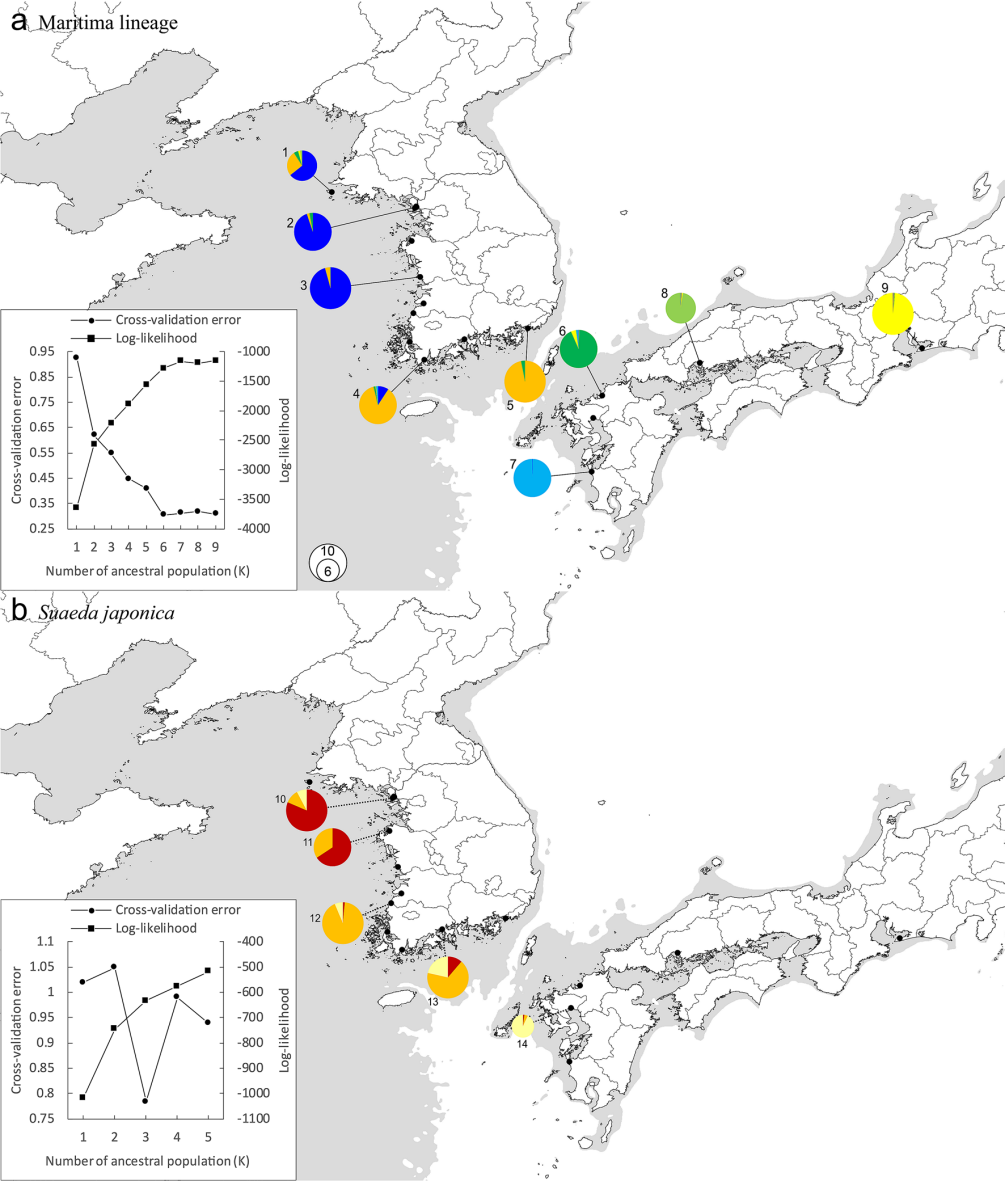
Admixture analysis from SNPs data and genotype in Maritima lineage and *S*. *japonica*. Background map was the same in Fig. 1.

Although a relatively small number of genome-wide SNPs was obtained from MIG-seq, it revealed relationships among the species. The PCoA scatter plot created from the first genome-wide SNP dataset (249 SNPs) including all populations revealed clear clustering in the Maritima lineage (*S*. *australis* in Korea and *S*. *maritima* in Japan), *S*. *japonica*, and the Heteroptera lineage (*S*. *maritima* in Korea and *S*. *heteroptera*) (Figs. 2, S2), although it was difficult to identify specific structuring in Admixture analysis with optimal K = 11 (Fig. S1). In lower K (3–5) with relatively low cross-validation error, *S*. *japonica* and Heteroptera lineage clusters together, and one or two populations of *S*. *japonica* admixed with a partial Maritima lineage (Fig. S1). In subsequent analyses based on genetic lineages identified in the PCoA, each lineage was analyzed separately. Admixture analysis of the Maritima lineage (112 SNPs) indicated that this lineage was largely formed by six clusters with optimal K = 6. The six clusters are separated into those from the western coast of Korea, the southern coast of Korea, and each population of Japan (Fig. 3). Each population in Japan was found to be distinct from other populations by ancestor lineages, indicating that there is little admixed ancestry in most populations. In PCoA analysis of the Maritima lineage, the scatter plot was in almost complete agreement with the Admixture results (Fig. S3). The genetic structure of *S*. *japonica* (74 SNPs) in Admixture seemed to reveal three clusters with optimal K = 3, which was slightly different from that of the Maritima lineage (Fig. 3). Each population had admixed ancestry. PCoA analysis of *S*. *japonica* indicated that all clusters were separated from each other (Fig. S3). Unlike the other two lineages, Admixture analysis of the Heteroptera lineage (84 SNPs) did not reveal significant genetic structures, with the best K = 1 (Fig. S4).

Genetic diversity was estimated from the first SNP dataset (249 SNPs) (Table 1). In the Maritima lineage, the mean values of expected heterozygosity and nucleotide diversity were 0.0641 and 0.0682, respectively. These values were lower in the Japanese populations than in the Korean populations. The mean values of expected heterozygosity and nucleotide diversity in *S*. *japonica* were 0.1366 and 0.1455, respectively. For the Heteroptera lineage, the mean values of expected heterozygosity and nucleotide diversity were 0.1605 and 0.1698, respectively.

### Phylogenetic analysis

We found obvious inconsistencies between phylogenetic trees based on cpDNA (*rpl32*-*trnL*) and nDNA (ITS) (Fig. S5). Based on a previous study^8^, species in this study were placed in a clade referred to as the ‘*S*. *maritima* group’ in the ITS tree. In the tree based on *rpl32*-*trnL* data, species are placed in a clade referred to as the ‘*S*. *corniculata* group’. In both phylogenetic trees, the Maritima lineage is comprised of a clade with higher supporting values (Fig. S5). However, the Heteroptera lineage and *S*. *japonica* each comprise a clade in the ITS tree (Fig. S5), while in the *rpl32*-*trnL* tree, most haplotypes of the Heteroptera lineage were placed in a clade with other species. Furthermore, haplotype C appears in the Heteroptera lineage, and *S*. *japonica* forms a clade related to the Maritima lineage. In addition, sequences from European *S*. *maritima* are separated from those of the Maritima lineage in East Asia in both phylogenetic trees. The *rpl32*-*trnL* sequence from the chloroplast genome of *S*. *salsa*^31^, which was obtained from a sample from Shangdong in China, is placed with *S*. *heteroptera*.

## Discussion

We investigated the genomic structure of four species traditionally listed for Korea, Japan, and NE China (*S*. *australis*, *S*. *maritima*, *S*. *japonica*, and *S*. *heteroptera*) using chloroplast DNA markers, nuclear ITS markers, and genome-wide SNP data*.* We found that they correspond to three lineages only, namely the Maritima lineage, Heteroptera lineage, and *S*. *japonica*, which disagrees with the circumscriptions given in regional floras.

**Figure 4:**
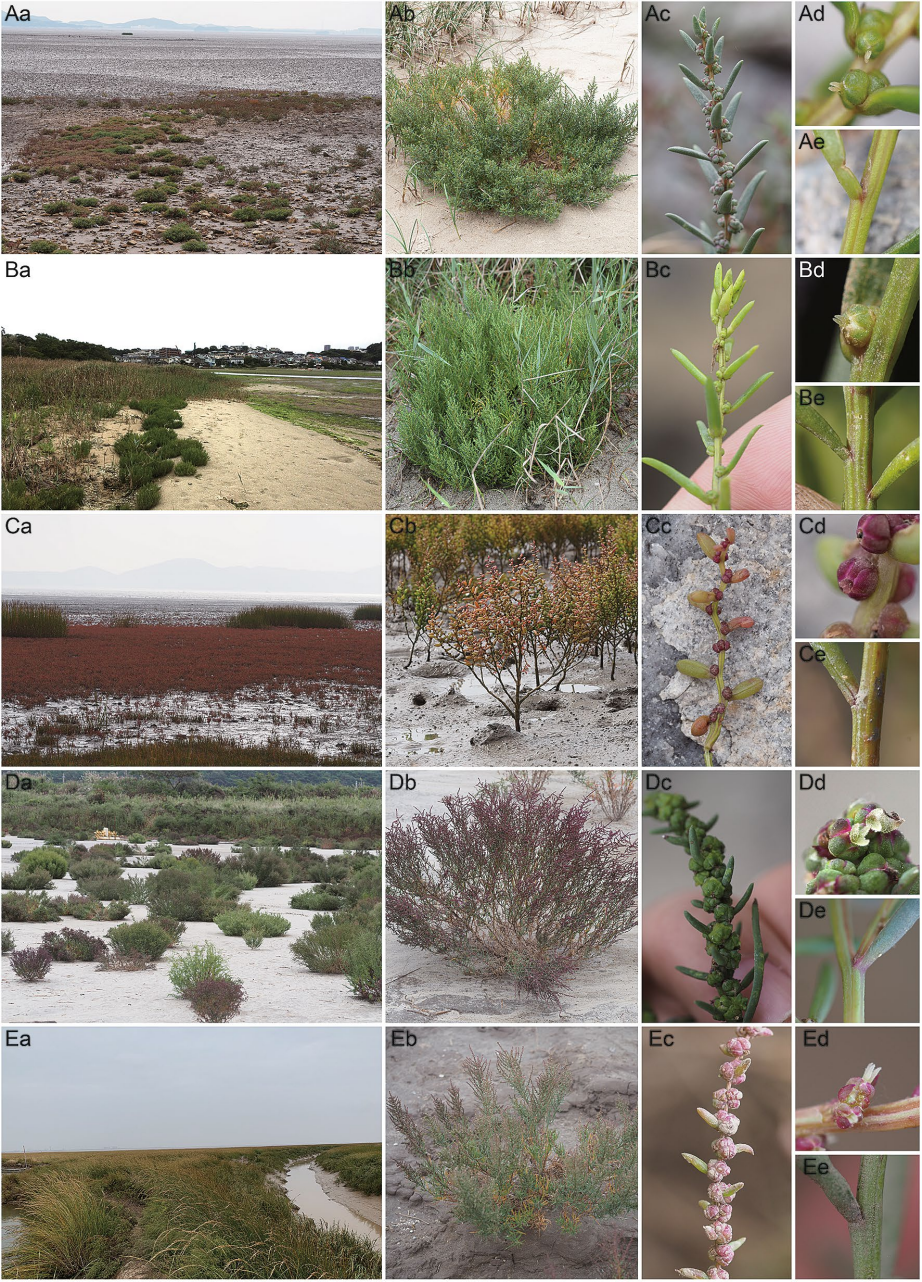
Habitat and morphological characters of *Suaeda* species in this study. (**A**) *Suaeda australis* in Korea, (**B**) *S*. *maritima* in Japan, (**C**) *S*. *japonica*, (**D**) *S*. *maritima* in Korea, (**E**) *S*. *heteroptera*, (a) habitat, (b) plant form, (c) inflorescence, (d) pistil, (e) base of leaf; (**A**,**B**) Maritima lineage and (**D**,**E**) Heteroptera lineage; Aa, c, Incheon-si, Republic of Korea; b, Seocheon-gun; d, e, Gunsan-si; Ba, b, Fukuoka-shi, Fukuoka Prefecture, Japan; c, Hiroshima-shi, Hiroshima Prefecture; d, e, Akune-shi, Kagoshima Prefecture; Ca-e, Incheon-si, Republic of Korea; Da, b, Boryeong-si, Republic of Korea; c, d, Gunsan-si; e, Incheon-si; Ea, c, Lianyungang-Shi, Jiangsu-Sheng, China; b, d, Yancheng-Shi; e, Nantong-Shi.

The Maritima lineage, which includes *S*. *australis* in Korea and *S*. *maritima* in Japan, is quite distinct from the Heteroptera lineage and *S*. *japonica* in the haplotype, ribotype network, and PCoA based on SNP data, but almost identical in molecular respect (Figs. 1, 2). This result allows for the statement *S*. *australis* does not occur in Korea, which was already questioned by Lee et al.^15^. The presence of *S*. *australis* in Korea was recorded based on its distribution in southern China and on morphological characters^14^. In our field research, morphological characters of *S*. *australis* in Korea, such as branched stems at the base and conspicuous leaf scars, were also observed in *S*. *maritima* in Japan (Fig. 4A,B). In our study, we found that the growth form of the Maritima lineage was affected by habitat conditions such as distance from the mouth of the river (which is related to salinity), the waterlogging cycle, and the density of individuals. Furthermore, the Maritima lineage in East Asia and *S*. *maritima* in Europe were separated in phylogenetic trees resulting from *rpl32*-*trnL* and ITS analyses (Fig. S5). This result is in agreement with Schütze^32^. *S*. *maritima* in East Asia was treated as *S*. *maritima* subsp. *asiatica* by Hara based on its karyotype (2n = 18), and generally decumbent or spreading-ascending branches^12,13^.

*Suaeda* *maritima* in Korea and *S*. *heteroptera* clustered together in the Heteroptera lineage in the PCoA analysis (Fig. 2). Their molecular identity is supported by our observations that in habitats outside of waterlogged areas, plants in this lineage have two or three stigmas and enlarged fruit perianths in Korea and China (Fig. 4D,E). This result is in agreement with Lee^20^ as well as Zhu and Clemants^2^. Even though Zhu and Clemants recognized *S*. *heteroptera* as a synonym of *S*. *salsa*, they documented the Korean distribution of *S*. *salsa*^2^. Also Lomonosova and Freitag^4^ noted relationship of *S*. *heteroptera* to *S*. *liaotungensis*, which described with having not enlarged perianth with regard to description of *S*. *maritma* in Korea, is in need of further study. These results suggest the need for a taxonomic revision of *S*. *maritima* and related species in East Asia, taking into consideration the taxonomic position of these species in Europe. This phenomenon, in which different names apply to the same lineages according to the region in which they are found, indicates that there is similar taxonomic confusion in the genus *Suaeda* as in the genus *Salicornia*, due to the absence of a global revision, the presence of phenotypic plasticity, and weak morphological differentiation^33^.

*Suaeda japonica* was clearly distinguished from the Heteroptera lineage in the PCoA analysis (Fig. 2), while it shared haplotype C and ribotypes with the Heteroptera lineage (Fig. 1). Although the placement of *S*. *japonica* in the genotype and phylogenetic tree is conflicting (Fig. S5), this species is morphologically clearly distinct from the Maritima and Heteroptera lineages (Fig. 4). *S*. *japonica* has an erect habit, a peculiar leaf shape, and turns reddish purple in autumn (Fig. 4C). We found that leaves of *S*. *japonica* are linear elliptic and semi-terete in the early flowering season (from June), but become fleshy and terete in the fruit season (September–October). The leaf base is articulate (Fig. 4Ce), and the withered leaves fall from the stem without leaving knobs. In an ecological respect, *S*. *japonica* occurs in unique habitats preferably on periodically waterlogged intertidal mud flats, which are assumed to be unsuitable for other *Suaeda* species. Based on the analysis of SNP data and morphological and ecological characters, we recognize *S*. *japonica* as a good species, although its relationship to the other *Suaeda* species, based on sequences of cpDNA and ITS, is unclear.

Analysis of the genetic structure of the Maritima lineage revealed that there are six clusters, which are divided into those present on the western coast of Korea, the southern coast of Korea, and four locations in Japan (Fig. 3a). These results are similar to those of a phylogeographic study on *S*. *malacosperma*^18^, which also revealed three clusters present on the western coast of Korea, the southern coast of Korea, and Japan^23^. Considering that the habitat of the Maritima lineage is near or in waterlogged areas, similar to the habitats in which *S*. *malacosperma* is found, it seems that the Maritima lineage experienced a post-glacial process similar to that experienced by *S*. *malacosperma*. This process can be summarized as range shifts associated with increasing sea levels resulting from climate change, which affected the genetic structure of plants in coastal regions. The six clusters (western coast of Korea, southern coast of Korea, and four locations in Japan) may represent recolonization routes after the glacial period. The Maritima lineage had a higher genetic diversity in the northern Korean populations than in the southern Japanese populations (Table 1). This does not correspond with the concept of ‘southern richness vs. northern purity’, which is a general genetic pattern shaped through climatic fluctuations and oscillations during the Quaternary period^34–36^. It seems that the loss of genetic diversity resulted from considerable range shifts, as also reported for *S*. *malacosperma*^18^. Another explanation for the genetic patterns in the Maritima lineage is that low genetic diversity may be the result of habitat loss due to anthropogenic coastal development. Based on personal observations, coastal habitats near well-developed areas in Japan were of poorer quality than coastal habitats in Korea.

The genetic structure of *S*. *japonica* differs from those of the Maritima lineage and *S*. *malacosperma* (Fig. 3b). It shows more admixture in each population than the Maritima lineage, and three clusters were largely similar to the Maritima lineage and *S*. *malacosperma*. As described above, the genetic structure of *S*. *japonica* could also have been affected by post- and interglacial processes. The slightly different genetic structure may be explained by very wide mud flat habitats, which can diminish the effect of genetic drifts by allowing the formation of one large population^37^. Furthermore, *S*. *heteroptera* has no significant genetic structure. This species may be affected by recent colonization into new habitats such as fish farms developed through human activity. Another explanation is that our regional sampling did not cover the entire distribution of *S*. *heteroptera*, which is widely distributed in eastern Eurasia. Our results suggest that the genetic structures of *Suaeda* species distributed in coastal regions are influenced by range shifts associated with climate change after the last glacial period. This is in accordance with the results of previous studies on coastal *S*. *malacosperma*^18^, *Triglochin* sp.^38^, and *Fucus* sp.^39^.

The phylogenetic relationship between *S*. *japonica* and the Heteroptera lineage based on cpDNA (*rpl32*-*trnL*) and ITS regions is somewhat unclear (Figs. 1, S5), even though these groups were found to be distinct based on PCoA analysis, morphological characters, and habitat (Figs. 2, 4). Recent phylogenetic study of Korean *Suaeda* species using four markers revealed that only single marker cannot distinguish those species, even *S*. *glauca* known as positioning in other section, except *S*. *malacosperma*^16^. In cpDNA markers and ITS, *S*. *japonica* consists of the same clade as *S*. *australis* and *S*. *maritima*, respectively^16^. This is concordant with our genotype and phylogenetic analysis (Fig. S5). We assume that both phylogenetic trees and genotype networks based on cpDNA and ITS suggest chloroplast capture from *S*. *japonica* into the Heteroptera lineage. Haplotype C of *S*. *japonica* is covered by haplotypes of the Heteroptera lineage, but haplotype C is more distant from most of the Heteroptera lineage haplotypes than from those of the Maritima lineage in the haplotype network by at least 40 steps, without a middle variety (Fig. 1a). This result is similar to the phylogenetic tree constructed using *rpl32*-*trnL*. To explain clear clustering in PCoA analysis based on SNPs, genotype networks, and the phylogenetic tree, it is plausible that hybridization between *S*. *japonica* as the maternal line and the Heteroptera lineage as the paternal line was followed by backcrossing with pollen of the paternal line. Discordance between cpDNA and ITS has been previously documented in phylogenetic studies of the genus *Suaeda*^8,32^. Brandt et al.^8^ found at least four cases of chloroplast capture and hybridization between distant *Suaeda* taxa in the Americas.

The wider distribution of haplotype C than the others in the Heteroptera lineage is interesting (Fig. 1a). There are two possible explanations for the distribution of haplotype C originating from chloroplast capture. The first is that the chloroplast capture event happened once, followed by the spreading of the haplotype. The second is that multiple chloroplast captures occurred in several locations. The latter is a simpler explanation for the distribution of haplotype C because it is difficult for haplotypes to spread into populations after a chloroplast capture event. We confirmed that plants of the Heteroptera lineage were almost always found around fish farms. Considering the habitats of plants in the Heteroptera lineage, and the distribution of haplotype C, the second explanation could be supported by an inferred process that followed the construction of fish farms in salt marsh habitats of *S*. *japonica*, followed by both restriction of *S*. *japonica* and colonization of plants of the Heteroptera lineage into these habitats, afterwards followed by hybridization between these populations and subsequent backcrossing. It is possible that there was an occasional chance movement of *S*. *japonica* into populations of *S*. *heteroptera*. Based on this theory, the distribution of haplotype C in the Heteroptera lineage in Korea (populations 15, 16, and 17), which are found near locations where *S*. *japonica* occurs, could be explained. Furthermore, the existence of haplotype C in population 19 may suggest a putative distribution of *S*. *japonica* in China.

Despite clear clustering of each species in the PCoA analysis based on SNPs (Fig. 2), the ITS sequences of both *S*. *japonica* and the Heteroptera lineage were almost identical (Figs. 1b, S5). In addition, three species in this study were found to have the same chromosome number (2n = 18)^12,40,41^. However, the morphological and ecological characters of *S*. *japonica* clearly indicate that it should be recognized as an individual species (Fig. 4). Based on the relationships between *S*. *japonica* and related species in the phylogenetic tree based on cpDNA, *S*. *japonica* may have experienced an independent evolution process. Because cpDNA has non-recombination traits and lower mutation levels than nuclear DNA, when we assume that *S*. *japonica* is an independent species, it is difficult to explain the closer genetic distance between *S*. *japonica* and *S*. *heteroptera* in ITS than *rpl32*-*trnL*.

Based on the karyotypes of the studied *Suaeda* species, and the fact that *S*. *japonica* (Figs. 1, 2) is closely related to the Maritima lineage based on cpDNA (which is inherited through the maternal line), and its relationship with the Heteroptera lineage shown through analysis of nDNA (which is also inherited through the paternal line), we hypothesize that *S*. *japonica* originated from homoploid hybrid speciation. This is also supported by the possibility of hybridization through chloroplast capture. In addition, admixed patterns among *S*. *japonica* and these lineages can fortify the validity of the hypothesis (Fig. S1). Homoploid hybrid speciation promoted by ecological selection has been described in several studies and reviews^42–44^. In a review by Gross and Rieseberg^42^, the authors explained that homoploid hybrid derivatives resulting from hybridization between parental species may have transgressive traits that can facilitate adaptation to extreme habitats where tolerance of the hybrids exceeds that of the parental species. Reproductive isolation of hybrid derivatives is achieved through the separation of habitats where hybrids and parental species occur. *S*. *japonica* has distinguishing inflated clavate leaves, and occurs in the middle of mudflats that are flooded by seawater twice a day. We hypothesize two scenarios for *S*. *japonica* speciation: (1) hybridization between the Heteroptera lineage as the paternal line and the Maritima lineage as the maternal line, and (2) hybridization between the Heteroptera lineage as the paternal line and other species related to the Maritima lineage. In haplotype network analysis, *S*. *japonica* was found to be more closely related to the Maritima lineage than to the Heteroptera lineage. This result supports the first hypothesis. However, the genetic distance of *S*. *japonica* haplotypes from the Maritima lineage is difficult to explain using the first hypothesis because cpDNA generally has a slower mutation rate than nuclear DNA. Therefore, if the maternal line was the Maritima lineage, the genetic distance would be closer to the Maritima lineage in cpDNA than to the Heteroptera lineage in nDNA. The second hypothesis assumes that the maternal line is an ancestor of, or another species associated with, the Maritima lineage. Our cpDNA analysis results could be explained by the fact that certain species accumulated mutations through independent evolutionary processes, and were derived from an ancestor that was shared with the Maritima lineage. Nevertheless, we cannot exclude the possibility of incomplete lineage sorting because only one marker sequence from each cpDNA and nDNA was used for phylogenetic analysis (Fig. S5). To confirm or refute homoploid hybrid speciation, further analysis with more markers and/or analysis at the genomic level is necessary.

In conclusion, this study significantly improves our understanding of the taxonomic relationships and evolution of *Suaeda* species in East Asia by using integrated genetic markers and genome-wide approaches. Our results may help resolve the problems surrounding *Suaeda* taxonomy in East Asia. Our results also suggest that the genetic structures of coastal *Suaeda* species in East Asia were affected by range shifts associated with climate change. We found that reticulate evolution could have played a role in the evolutionary processes of the genus *Suaeda*.

## Methods

### Sampling and sequencing

We collected leaf samples from 192 individual plants, including partial voucher specimens, belonging to 19 populations of *S*. *maritima*, *S*. *australis*, *S*. *japonica,* and *S*. *heteroptera*, growing along the coastal regions of Korea, Japan, and China from 2016 to 2019 (Tables S1 and S2). We also sampled *S*. *glauca* for use as an outgroup in our genetic analyses (Tables S1 and S2). Leaf samples were collected from plants during the fruiting season, and sampled plants were located at least 5–10 m from each other. Collected samples were dried in silica gel and stored at room temperature. Genomic DNA (gDNA) was extracted using the DNeasy Plant Mini Kit (Qiagen, Seoul, Korea), and the concentration of each extracted gDNA sample was measured using a NanoDrop Spectrophotometer (NanoDrop Technologies, Wilmington, DE, USA) before PCR amplification and MIG-seq.

Sequences of the two non-coding regions of cpDNA (*rpl32*-*trnL*^45^ and *trnH*-*psbA*^46^) and one region of nDNA (internal transcribed spacer (ITS)^47^) were amplified. These regions were chosen following the methods of Park et al.^18^ and Lee et al.^15^. Because of the low genetic diversity found in a phylogeographic study of *S*. *malacosperma*^18^, we used 96 samples (five per population) of the 192 samples, including *S*. *glauca*. We performed PCR using a GeneAmp PCR System 2720 Thermal Cycler (Applied Biosystems, Foster City, CA, USA). PCR conditions were as described by Park et al.^18^. After PCR products were checked by visualizing them on 2% agarose gels, they were purified with an MG PCR Purification kit (MGmed, Seoul, Korea), and sequenced with the ABI PRISM 3730XL Analyzer, using the BigDye Terminator v3.1 Cycle Sequencing Kits (Applied Biosystems). The sequences were deposited in the GenBank database (Table S2).

### Genotype network and phylogenetic analysis

Sequences of the collected *Suaeda* samples from Korea, Japan, and China were aligned using Muscle^48^ and Geneious 10.2.6 (Biomatters Ltd., Auckland, NZ). For consensus haplotypes, the sequence of each individual was concatenated from those of the *rpl32*-*trnL* and *psbA*-*trnH* regions. Both haplotypes and ribotypes were identified from aligned sequences completed using manual adjustments. Detected inversion, indel, and simple sequence repeats were dealt with using one-point mutation steps in genotype network analysis. The relatedness of each haplotype and ribotype was expressed in a statistical parsimony network, generated by the program TCS version 1.21^27^.

In order to describe phylogenetic relationships between the species evaluated in this study within the genus *Suaeda* sect. Brezia, we obtained sequence data of related *Suaeda* species and used sequence data of *Bienertia sinuspersici* Akhani obtained from GenBank as an outgroup (Fig. S5). We used both genotypes of each species entity and filtered out the duplicates. Considering the difficulty in identifying *Suaeda* species due to the lack of diagnostic characters, we used the sequences obtained in a previous phylogenetic study^8^. The *rpl32*-*trnL* regions of the documented chloroplast genomes were also used^31,49^. Maximum likelihood trees based on *rpl32*-*trnL* and ITS data were constructed using W-IQ-TREE^50^, based on user-friendly web servers for IQ-TREE^51^. Each aligned sequence dataset was tested to find the best-fit model by using W-IQ-TREE with the Akaike criterion and new model selection procedures. GTR + R2 + F and GTR + G4 + F were confirmed as best-fit models for *rpl32*-*trnL* and ITS, respectively. Maximum likelihood analysis of both aligned sequences was performed with default settings in W-IQ-TREE.

### Obtaining SNPs from MIG-seq

We obtained genome-wide SNPs by using MIG-seq methods following the protocols of Park et al.^18^, without a dark cycle. In summary, the first PCR was performed using designated primers to target inter-simple sequence repeats (ISSR), followed by a second PCR using the amplicons from the first PCR amplicons to index each sample. After this two-step PCR, each amplicon was pooled and purified with a QIAquick PCR Purification Kit (Qiagen, Seoul, Korea). The pooled sample was size-selected in the range of 350–600 bp using the BluePippin system (Sage Science, Beverly, USA), and was sequenced using a 2 × 150 bp paired-end on the Illumina MiSeq platform (Illumina, San Diego, CA, USA), using a MiSeq Reagent kit v2 (Illumina) at LAS (Gimpo, Republic of Korea).

In the obtained NGS data, the adapter and anchor of each primer was removed from the sequenced reads, and overlapping and low-quality reads were excluded using the FASTX Toolkit 0.0.14 (http://hannonlab.cshl.edu/fastx_toolkit/) and TagDust 1.12^52^. We subsequently analyzed the processed reads using Stacks v1.48^53^. The stacks were assembled de novo and piled with the option of the minimum depth coverage set at m = 3, and the maximum distance set at M = 2. The other options were set at default settings. When the loci catalog of stacks was produced, two mismatches were allowed. The SNP dataset for population genetics was created using the ‘population’ command in the Stacks program, with the following criteria: at least 75% of a locus shared within a population (−r 0.75), a population where a locus had to be present (− p 1), minimum minor allele frequency of 0.05 (–min_maf 0.05), maximum observed heterozygosity of 0.95 (–max_obs_het 0.95), and including only the first SNP per locus (–wrire_single_snp option). SNP datasets were produced for two-level analysis of genetic structure. The first level consisted of all populations, and the second comprised populations of each of the genetic lineages identified from the first level of analysis.

### Analysis of population genetics

To compare cpDNA and nDNA markers, genetic diversity and structure were analyzed from genome-wide SNP data. Genetic diversity was estimated using the population command in Stacks. Principal Coordinates Analysis (PCoA) was performed using the first SNP dataset to identify the taxonomic relationships using GenAlEx 6.51b2^29,30^ (https://biology-assets.anu.edu.au/GenAlEx/). The ancestry of each sample was estimated using Admixture 1.3.0^54^ with tenfold cross-validation. The number of ancestors in the range 1 to the number of populations was tested using both SNP datasets, and that with the lowest cross-validation error was selected. In addition, SNP data sets with other parameters (− r = 0.5, 0.75; − p = 1, 2) were produced and tested to confirm the consistency of the population genetics results.

## Supplementary information

Supplementary Information.
