# Supplementary material for: Insights into genomic structure and evolutionary processes of coastal Suaeda species in East Asia using cpDNA, nDNA, and genome-wide SNPs
Source: Sci Rep. 2020 Dec 1;10:20950. doi: 10.1038/s41598-020-78041-7 (PMC7708624; doi:10.1038/s41598-020-78041-7)
Supplement: Supplementary file 1 — Supplementary Information. [file 41598_2020_78041_MOESM1_ESM.pdf]

Supplementary Information

Insights into genomic structure and evolutionary processes of coastal *Suaeda* species in East Asia using cpDNA, nDNA, and genome-wide SNPs

Jong-Soo Park, Dong-Pil Jin and Byoung-Hee Choi*

Department of Biological Sciences, Inha University, Incheon, 22212, Republic of Korea

Correspondence and requests for materials should be addressed to B.-H.C.

(email:bhchoi@inha.ac.kr)

Table S1. Localities of *Suaeda australis*, *S. maritima*, *S. japonica* and *S. heteroptera* in Korea, Japan, and China

species	No.	Locality	Coordinate	Date
Suaeda australis in Korea	1	Isl. Baengnyeong Baengnyeong-myeon, Ongjin-gun, Incheon, Republic of Korea	N 37.95, E 124.72	Oct. 14. 2018
	2	Sagi-ri, Hwado-myeon, Ganghwa-gun, Incheon, Republic of Korea	N 37.60, E 126.47	Oct. 16. 2019
	3	Wolho-ri, Seo-myeon, Seocheon-gun, Chungcheongnam-do, Republic of Korea	N 36.13, E 126.56	Sep. 30. 2016
	4	Waryong-ri, Bukpyeong-myeon, Haenam-gun, Jeollanam-do, Republic of Korea	N 34.42, E 126.65	Oct. 14. 2016
	5	Isl. Gaduk, Dongseon-dong, Gangseo-gu, Busan, Republic of Korea	N 35.05, E 128.83	Oct. 07. 2019
S. maritima in Japan	6	Wajiro beach, Fukuoka-shi, Fukuoka Prefecture, Japan	N 33.68, E 130.43	Sep. 28. 2017
	7	Wakimoto Beach, Akune-shi, Kagoshima Prefecture, Japan	N 32.06, E 130.20	Sep. 29. 2017
	8	Riverside, Fukushima-cho, Hiroshima-shi, Hiroshima Prefecture, Japan	N 34.39, E 132.43	Sep. 27. 2017
	9	Aichi Prefecture, Japan	N 34.64, E 137.13	Sep. 10. 2017
S. japonica	10	Dongmak-ri, Hwado-myeon, Ganghwa-gun, Incheon, Republic of Korea	N 37.59, E 126.44	Oct. 21. 2016
	11	Doseong-ri, Jigok-myeon, Seosan-si, Chungcheongnam-do, Republic of Korea	N 36.89, E 126.41	Oct. 01. 2016
	12	Beopseong-myeon, Yeonggwang-gun, Jeollanam-do, Republic of Korea	N 35.35, E 126.44	Oct. 23. 2019
	13	Byeollyang-myeon, Suncheon-si, Jeollanam-do, Republic of Korea	N 34.86, E 127.49	Oct. 14. 2016
	14	Higatayaka Park, Saga-shi, Saga Prefecture, Japan	N 33.19, E 130.20	Sep. 24. 2017
S. maritima in Japan	15	Sagi-ri, Hwado-myeon, Ganghwa-gun, Incheon, Republic of Korea	N 37.61, E 126.46	Oct. 16. 2019
	16	Boan-myeon, Buan-gun, Jeollabuk-do, Republic of Korea	N 35.59, E 126.64	Oct. 23. 2016
	17	Janggam-ri, Aphae-eup, Sinan-gun, Jeollanam-do, Republic of Korea	N 34.81, E 126.34	Oct. 19. 2019
S. heteroptera in China	18	Around Xian port, Qidong Shi, Nantong Shi, Jiangsu Sheng, China	N 31.85, E 121.86	Oct. 10. 2019
	19	Guanyun, Lianyungang Shi, Jiangsu Sheng, China	N 34.52, E 119.67	Oct. 12. 2019
S. glauca		Songnim-ri, Masan-myeon, Seocheon-gun, Chungcheongnam-do, Republic of Korea	N 36.02, E 126.66	Sep. 29. 2016

Table S2. Sample information

Species	Locality	Date	Collector	Voucher No.	Sample ID	ITS	Haplotype	ITS Genbank Accession	rpl32-trnL Genbank Accession	psbA-trnH Genbank Accession	Lineage
Suaeda australis (R. Br.) Moq.	Sa-got Beach, Baengnyeong-myeon, Ongjin-gun, Incheon, Republic of Korea	2018-10-14	J. W. Park	P1810151	ICB1A	A	A	MT875093	MT873670	MT873766	Maritima lineage
Suaeda australis (R. Br.) Moq.	Sa-got Beach, Baengnyeong-myeon, Ongjin-gun, Incheon, Republic of Korea	2018-10-14	J. W. Park	P1810152	ICB2A						Maritima lineage
Suaeda australis (R. Br.) Moq.	Sa-got Beach, Baengnyeong-myeon, Ongjin-gun, Incheon, Republic of Korea	2018-10-14	J. W. Park	P1810154	ICB4A	A	A	MT875094	MT873671	MT873767	Maritima lineage
Suaeda australis (R. Br.) Moq.	Sa-got Beach, Baengnyeong-myeon, Ongjin-gun, Incheon, Republic of Korea	2018-10-14	J. W. Park	P1810155	ICB5A						Maritima lineage
Suaeda australis (R. Br.) Moq.	Sa-got Beach, Baengnyeong-myeon, Ongjin-gun, Incheon, Republic of Korea	2018-10-14	J. W. Park	P1810156	ICB6A	A	A	MT875095	MT873672	MT873768	Maritima lineage
Suaeda australis (R. Br.) Moq.	Sa-got Beach, Baengnyeong-myeon, Ongjin-gun, Incheon, Republic of Korea	2018-10-14	J. W. Park	P1810157	ICB7A						Maritima lineage
Suaeda australis (R. Br.) Moq.	Sa-got Beach, Baengnyeong-myeon, Ongjin-gun, Incheon, Republic of Korea	2018-10-14	J. W. Park	P1810159	ICB9A	A	A	MT875096	MT873673	MT873769	Maritima lineage
Suaeda australis (R. Br.) Moq.	Sa-got Beach, Baengnyeong-myeon, Ongjin-gun, Incheon, Republic of Korea	2018-10-14	J. W. Park	P1810160	ICB10A	A	A	MT875097	MT873674	MT873770	Maritima lineage
Suaeda australis (R. Br.) Moq.	Beach, Sagi-ri, Hwado-myeon, Ganghwa-gun, Incheon, Republic of Korea	2019-10-16	J. S. Park	1910301	GH1A	A	A	MT875063	MT873650	MT873746	Maritima lineage
Suaeda australis (R. Br.) Moq.	Beach, Sagi-ri, Hwado-myeon, Ganghwa-gun, Incheon, Republic of Korea	2019-10-16	J. S. Park	1910303	GH3A						Maritima lineage
Suaeda australis (R. Br.) Moq.	Beach, Sagi-ri, Hwado-myeon, Ganghwa-gun, Incheon, Republic of Korea	2019-10-16	J. S. Park	1910304	GH4A	A	A	MT875066	MT873653	MT873749	Maritima lineage
Suaeda australis (R. Br.) Moq.	Beach, Sagi-ri, Hwado-myeon, Ganghwa-gun, Incheon, Republic of Korea	2019-10-16	J. S. Park	1910306	GH6A						Maritima lineage
Suaeda australis (R. Br.) Moq.	Beach, Sagi-ri, Hwado-myeon, Ganghwa-gun, Incheon, Republic of Korea	2019-10-16	J. S. Park	1910308	GH8A	A	A	MT875068	MT873655	MT873751	Maritima lineage
Suaeda australis (R. Br.) Moq.	Beach, Sagi-ri, Hwado-myeon, Ganghwa-gun, Incheon, Republic of Korea	2019-10-16	J. S. Park	1910310	GH10A						Maritima lineage
Suaeda australis (R. Br.) Moq.	Seondu-ri port, Gilsang-myeon, Ganghwa-gun, Incheon, Republic of Korea	2019-10-16	J. S. Park	1910312	GH12A						Maritima lineage
Suaeda australis (R. Br.) Moq.	Seondu-ri port, Gilsang-myeon, Ganghwa-gun, Incheon, Republic of Korea	2019-10-16	J. S. Park	1910316	GH16A	A	A	MT875072	MT873659	MT873755	Maritima lineage
Suaeda australis (R. Br.) Moq.	Seondu-ri port, Gilsang-myeon, Ganghwa-gun, Incheon, Republic of Korea	2019-10-16	J. S. Park	1910318	GH18A						Maritima lineage
Suaeda australis (R. Br.) Moq.	Seondu-ri port, Gilsang-myeon, Ganghwa-gun, Incheon, Republic of Korea	2019-10-16	J. S. Park	1910320	GH20A	A	A	MT875075	MT873662	MT873758	Maritima lineage
Suaeda australis (R. Br.) Moq.	River mouth around, Wolho-ri, Seo-myeon, Seocheon-gun, Chungcheongnam-do, Republic of Korea	2016-09-30	J. S. Park	1609151	seo1M	A	A	MT875119	MT873686	MT873782	Maritima lineage
Suaeda australis (R. Br.) Moq.	River mouth around, Wolho-ri, Seo-myeon, Seocheon-gun, Chungcheongnam-do, Republic of Korea	2016-09-30	J. S. Park	1609154	seo4M						Maritima lineage
Suaeda australis (R. Br.) Moq.	River mouth around, Wolho-ri, Seo-myeon, Seocheon-gun, Chungcheongnam-do, Republic of Korea	2016-09-30	J. S. Park	1609158	seo8M	A	A	MT875120	MT873687	MT873783	Maritima lineage
Suaeda australis (R. Br.) Moq.	River mouth around, Wolho-ri, Seo-myeon, Seocheon-gun, Chungcheongnam-do, Republic of Korea	2016-09-30	J. S. Park	1609161	seo11M						Maritima lineage
Suaeda australis (R. Br.) Moq.	River mouth around, Wolho-ri, Seo-myeon, Seocheon-gun, Chungcheongnam-do, Republic of Korea	2016-09-30	J. S. Park	1609164	seo14M	A	A	MT875121	MT873688	MT873784	Maritima lineage
Suaeda australis (R. Br.) Moq.	River mouth around, Wolho-ri, Seo-myeon, Seocheon-gun, Chungcheongnam-do, Republic of Korea	2016-09-30	J. S. Park	1609166	seo16M						Maritima lineage
Suaeda australis (R. Br.) Moq.	River mouth around, Wolho-ri, Seo-myeon, Seocheon-gun, Chungcheongnam-do, Republic of Korea	2016-09-30	J. S. Park	1609167	seo17M						Maritima lineage
Suaeda australis (R. Br.) Moq.	River mouth around, Wolho-ri, Seo-myeon, Seocheon-gun, Chungcheongnam-do, Republic of Korea	2016-09-30	J. S. Park	1609170	seo20M						Maritima lineage
Suaeda australis (R. Br.) Moq.	River mouth around, Wolho-ri, Seo-myeon, Seocheon-gun, Chungcheongnam-do, Republic of Korea	2016-09-30	J. S. Park	1609172	seo22M	A	A	MT875122	MT873689	MT873785	Maritima lineage
Suaeda australis (R. Br.) Moq.	River mouth around, Wolho-ri, Seo-myeon, Seocheon-gun, Chungcheongnam-do, Republic of Korea	2016-09-30	J. S. Park	1609174	seo24M						Maritima lineage
Suaeda australis (R. Br.) Moq.	River mouth around, Wolho-ri, Seo-myeon, Seocheon-gun, Chungcheongnam-do, Republic of Korea	2016-09-30	J. S. Park	1609177	seo27M	A	A	MT875123	MT873690	MT873786	Maritima lineage
Suaeda australis (R. Br.) Moq.	Estuary of Dong-hae stream, Waryong-ri, Bukpyeong-myeon, Haenam-gun, Jeollanam-do, Republic of Korea	2016-10-14	J. S. Park	1610071	HN1A	A	A	MT875083	MT873665	MT873761	Maritima lineage
Suaeda australis (R. Br.) Moq.	Estuary of Dong-hae stream, Waryong-ri, Bukpyeong-myeon, Haenam-gun, Jeollanam-do, Republic of Korea	2016-10-14	J. S. Park	1610074	HN4A						Maritima lineage
Suaeda australis (R. Br.) Moq.	Estuary of Dong-hae stream, Waryong-ri, Bukpyeong-myeon, Haenam-gun, Jeollanam-do, Republic of Korea	2016-10-14	J. S. Park	1610077	HN7A	A	A	MT875084	MT873666	MT873762	Maritima lineage
Suaeda australis (R. Br.) Moq.	Estuary of Dong-hae stream, Waryong-ri, Bukpyeong-myeon, Haenam-gun, Jeollanam-do, Republic of Korea	2016-10-14	J. S. Park	1610078	HN8A						Maritima lineage
Suaeda australis (R. Br.) Moq.	Estuary of Dong-hae stream, Waryong-ri, Bukpyeong-myeon, Haenam-gun, Jeollanam-do, Republic of Korea	2016-10-14	J. S. Park	1610080	HN10A						Maritima lineage
Suaeda australis (R. Br.) Moq.	Estuary of Dong-hae stream, Waryong-ri, Bukpyeong-myeon, Haenam-gun, Jeollanam-do, Republic of Korea	2016-10-14	J. S. Park	1610082	HN12A	A	A	MT875085	MT873667	MT873763	Maritima lineage
Suaeda australis (R. Br.) Moq.	Estuary of Dong-hae stream, Waryong-ri, Bukpyeong-myeon, Haenam-gun, Jeollanam-do, Republic of Korea	2016-10-14	J. S. Park	1610083	HN13A						Maritima lineage
Suaeda australis (R. Br.) Moq.	Estuary of Dong-hae stream, Waryong-ri, Bukpyeong-myeon, Haenam-gun, Jeollanam-do, Republic of Korea	2016-10-14	J. S. Park	1610086	HN16A	A	A	MT875086	MT873668	MT873764	Maritima lineage
Suaeda australis (R. Br.) Moq.	Estuary of Dong-hae stream, Waryong-ri, Bukpyeong-myeon, Haenam-gun, Jeollanam-do, Republic of Korea	2016-10-14	J. S. Park	1610089	HN19A						Maritima lineage
Suaeda australis (R. Br.) Moq.	Estuary of Dong-hae stream, Waryong-ri, Bukpyeong-myeon, Haenam-gun, Jeollanam-do, Republic of Korea	2016-10-14	J. S. Park	1610091	HN21A	A	A	MT875087	MT873669	MT873765	Maritima lineage
Suaeda australis (R. Br.) Moq.	Seashore, Isl. Gadeok, Dongseon-dong, Gangseo-gu, Busan, Republic of Korea	2019-10-07	J. S. Park	1910091	BSA1A	A	A	MT875048	MT873640	MT873736	Maritima lineage
Suaeda australis (R. Br.) Moq.	Seashore, Isl. Gadeok, Dongseon-dong, Gangseo-gu, Busan, Republic of Korea	2019-10-07	J. S. Park	1910092	BSA2A						Maritima lineage
Suaeda australis (R. Br.) Moq.	Seashore, Isl. Gadeok, Dongseon-dong, Gangseo-gu, Busan, Republic of Korea	2019-10-07	J. S. Park	1910093	BSA3A	A	A	MT875049	MT873641	MT873737	Maritima lineage
Suaeda australis (R. Br.) Moq.	Seashore, Isl. Gadeok, Dongseon-dong, Gangseo-gu, Busan, Republic of Korea	2019-10-07	J. S. Park	1910095	BSA5A						Maritima lineage
Suaeda australis (R. Br.) Moq.	Seashore, Isl. Gadeok, Dongseon-dong, Gangseo-gu, Busan, Republic of Korea	2019-10-07	J. S. Park	1910096	BSA6A						Maritima lineage

Suaeda australis (R. Br.) Moq.	Seashore, Isl. Gadeok, Dongseon-dong, Gangseo-gu, Busan, Republic of Korea	2019-10-07	J. S. Park	1910097	BSA7A	A	A	MT875050	MT873642	MT873738	Maritima lineage
Suaeda australis (R. Br.) Moq.	Seashore, Isl. Gadeok, Dongseon-dong, Gangseo-gu, Busan, Republic of Korea	2019-10-07	J. S. Park	1910098	BSA8A						Maritima lineage
Suaeda australis (R. Br.) Moq.	Seashore, Isl. Gadeok, Dongseon-dong, Gangseo-gu, Busan, Republic of Korea	2019-10-07	J. S. Park	1910099	BSA9A	A	A	MT875051	MT873643	MT873739	Maritima lineage
Suaeda australis (R. Br.) Moq.	Seashore, Isl. Gadeok, Dongseon-dong, Gangseo-gu, Busan, Republic of Korea	2019-10-07	J. S. Park	1910101	BSA11A						Maritima lineage
Suaeda australis (R. Br.) Moq.	Seashore, Isl. Gadeok, Dongseon-dong, Gangseo-gu, Busan, Republic of Korea	2019-10-07	J. S. Park	1910102	BSA12A						Maritima lineage
Suaeda australis (R. Br.) Moq.	Seashore, Isl. Gadeok, Dongseon-dong, Gangseo-gu, Busan, Republic of Korea	2019-10-07	J. S. Park	1910103	BSA13A	A	B	MT875052	MT873644	MT873740	Maritima lineage
Suaeda maritima (L.) Dumort.	Wajiro beach, Fukuoka-shi, Fukuoka Prefecture, Japan	2017-09-28	J. S. Park & J. W. Park	-	FO2M	B	A	MT875058	MT873625	MT873721	Maritima lineage
Suaeda maritima (L.) Dumort.	Wajiro beach, Fukuoka-shi, Fukuoka Prefecture, Japan	2017-09-28	J. S. Park & J. W. Park	-	FO4M						Maritima lineage
Suaeda maritima (L.) Dumort.	Wajiro beach, Fukuoka-shi, Fukuoka Prefecture, Japan	2017-09-28	J. S. Park & J. W. Park	-	FO5M	B	A	MT875059	MT873626	MT873722	Maritima lineage
Suaeda maritima (L.) Dumort.	Wajiro beach, Fukuoka-shi, Fukuoka Prefecture, Japan	2017-09-28	J. S. Park & J. W. Park	-	FO6M						Maritima lineage
Suaeda maritima (L.) Dumort.	Wajiro beach, Fukuoka-shi, Fukuoka Prefecture, Japan	2017-09-28	J. S. Park & J. W. Park	-	FO7M	A	A	MT875060	MT873627	MT873723	Maritima lineage
Suaeda maritima (L.) Dumort.	Wajiro beach, Fukuoka-shi, Fukuoka Prefecture, Japan	2017-09-28	J. S. Park & J. W. Park	-	FO8M						Maritima lineage
Suaeda maritima (L.) Dumort.	Wajiro beach, Fukuoka-shi, Fukuoka Prefecture, Japan	2017-09-28	J. S. Park & J. W. Park	-	FO9M	B	A	MT875061	MT873628	MT873724	Maritima lineage
Suaeda maritima (L.) Dumort.	Wajiro beach, Fukuoka-shi, Fukuoka Prefecture, Japan	2017-09-28	J. S. Park & J. W. Park	-	FO10M						Maritima lineage
Suaeda maritima (L.) Dumort.	Wajiro beach, Fukuoka-shi, Fukuoka Prefecture, Japan	2017-09-28	J. S. Park & J. W. Park	-	FO12M						Maritima lineage
Suaeda maritima (L.) Dumort.	Wajiro beach, Fukuoka-shi, Fukuoka Prefecture, Japan	2017-09-28	J. S. Park & J. W. Park	-	FO13M	B	A	MT875062	MT873629	MT873725	Maritima lineage
Suaeda maritima (L.) Dumort.	Wakimoto Beach, Akune-shi, Kagoshima Prefecture, Japan	2017-09-29	J. S. Park & J. W. Park	-	AKN1M	A	A	MT875043	MT873620	MT873716	Maritima lineage
Suaeda maritima (L.) Dumort.	Wakimoto Beach, Akune-shi, Kagoshima Prefecture, Japan	2017-09-29	J. S. Park & J. W. Park	-	AKN2M						Maritima lineage
Suaeda maritima (L.) Dumort.	Wakimoto Beach, Akune-shi, Kagoshima Prefecture, Japan	2017-09-29	J. S. Park & J. W. Park	-	AKN4M						Maritima lineage
Suaeda maritima (L.) Dumort.	Wakimoto Beach, Akune-shi, Kagoshima Prefecture, Japan	2017-09-29	J. S. Park & J. W. Park	-	AKN5M	A	A	MT875044	MT873621	MT873717	Maritima lineage
Suaeda maritima (L.) Dumort.	Wakimoto Beach, Akune-shi, Kagoshima Prefecture, Japan	2017-09-29	J. S. Park & J. W. Park	-	AKN6M						Maritima lineage
Suaeda maritima (L.) Dumort.	Wakimoto Beach, Akune-shi, Kagoshima Prefecture, Japan	2017-09-29	J. S. Park & J. W. Park	-	AKN7M	A	A	MT875045	MT873622	MT873718	Maritima lineage
Suaeda maritima (L.) Dumort.	Wakimoto Beach, Akune-shi, Kagoshima Prefecture, Japan	2017-09-29	J. S. Park & J. W. Park	-	AKN8M						Maritima lineage
Suaeda maritima (L.) Dumort.	Wakimoto Beach, Akune-shi, Kagoshima Prefecture, Japan	2017-09-29	J. S. Park & J. W. Park	-	AKN10M	A	A	MT875046	MT873623	MT873719	Maritima lineage
Suaeda maritima (L.) Dumort.	Wakimoto Beach, Akune-shi, Kagoshima Prefecture, Japan	2017-09-29	J. S. Park & J. W. Park	1709913	AKN13M						Maritima lineage
Suaeda maritima (L.) Dumort.	Wakimoto Beach, Akune-shi, Kagoshima Prefecture, Japan	2017-09-29	J. S. Park & J. W. Park	-	AKN14M	A	A	MT875047	MT873624	MT873720	Maritima lineage
Suaeda maritima (L.) Dumort.	Riverside, Fukushima-cho, Hiroshima-shi, Hiroshima Prefecture, Japan	2017-09-27	J. S. Park & J. W. Park	1709501	HS1M	A	A	MT875088	MT873630	MT873726	Maritima lineage
Suaeda maritima (L.) Dumort.	Riverside, Fukushima-cho, Hiroshima-shi, Hiroshima Prefecture, Japan	2017-09-27	J. S. Park & J. W. Park	-	HS2M						Maritima lineage
Suaeda maritima (L.) Dumort.	Riverside, Fukushima-cho, Hiroshima-shi, Hiroshima Prefecture, Japan	2017-09-27	J. S. Park & J. W. Park	-	HS3M	A	A	MT875089	MT873631	MT873727	Maritima lineage
Suaeda maritima (L.) Dumort.	Riverside, Fukushima-cho, Hiroshima-shi, Hiroshima Prefecture, Japan	2017-09-27	J. S. Park & J. W. Park	-	HS4M						Maritima lineage
Suaeda maritima (L.) Dumort.	Riverside, Fukushima-cho, Hiroshima-shi, Hiroshima Prefecture, Japan	2017-09-27	J. S. Park & J. W. Park	-	HS5M	A	A	MT875090	MT873632	MT873728	Maritima lineage
Suaeda maritima (L.) Dumort.	Riverside, Fukushima-cho, Hiroshima-shi, Hiroshima Prefecture, Japan	2017-09-27	J. S. Park & J. W. Park	1709506	HS6M						Maritima lineage
Suaeda maritima (L.) Dumort.	Riverside, Fukushima-cho, Hiroshima-shi, Hiroshima Prefecture, Japan	2017-09-27	J. S. Park & J. W. Park	1709507	HS7M	A	A	MT875091	MT873633	MT873729	Maritima lineage
Suaeda maritima (L.) Dumort.	Riverside, Fukushima-cho, Hiroshima-shi, Hiroshima Prefecture, Japan	2017-09-27	J. S. Park & J. W. Park	-	HS8M	A	A	MT875092	MT873634	MT873730	Maritima lineage
Suaeda maritima (L.) Dumort.	Dahara-shi, Aichi Prefecture, Japan	2017-09-10	Koji Takayama	2-3	AIC3M	A	A	MT875038	MT873615	MT873711	Maritima lineage
Suaeda maritima (L.) Dumort.	Dahara-shi, Aichi Prefecture, Japan	2017-09-10	Koji Takayama	2-6	AIC6M						Maritima lineage
Suaeda maritima (L.) Dumort.	Dahara-shi, Aichi Prefecture, Japan	2017-09-10	Koji Takayama	2-9	AIC9M	A	A	MT875039	MT873616	MT873712	Maritima lineage
Suaeda maritima (L.) Dumort.	Dahara-shi, Aichi Prefecture, Japan	2017-09-10	Koji Takayama	2-13	AIC13M						Maritima lineage
Suaeda maritima (L.) Dumort.	Dahara-shi, Aichi Prefecture, Japan	2017-09-10	Koji Takayama	2-14	AIC14M	A	A	MT875040	MT873617	MT873713	Maritima lineage
Suaeda maritima (L.) Dumort.	Dahara-shi, Aichi Prefecture, Japan	2017-09-10	Koji Takayama	2-15	AIC15M						Maritima lineage
Suaeda maritima (L.) Dumort.	Dahara-shi, Aichi Prefecture, Japan	2017-09-10	Koji Takayama	2-19	AIC19M						Maritima lineage
Suaeda maritima (L.) Dumort.	Dahara-shi, Aichi Prefecture, Japan	2017-09-10	Koji Takayama	3-1	AIC31M	A	A	MT875041	MT873618	MT873714	Maritima lineage
Suaeda maritima (L.) Dumort.	Dahara-shi, Aichi Prefecture, Japan	2017-09-10	Koji Takayama	3-2	AIC32M						Maritima lineage
Suaeda maritima (L.) Dumort.	Dahara-shi, Aichi Prefecture, Japan	2017-09-10	Koji Takayama	3-5	AIC35M						Maritima lineage
Suaeda maritima (L.) Dumort.	Dahara-shi, Aichi Prefecture, Japan	2017-09-10	Koji Takayama	3-7	AIC37M	A	A	MT875042	MT873619	MT873715	Maritima lineage
Suaeda japonica Makino	Dongmak-ri, Hwado-myeon, Ganghwa-gun, Incheon, Republic of Korea	2016-10-21	J. S. Park	1610101	GH1J	C	C	MT875064	MT873651	MT873747	
Suaeda japonica Makino	Dongmak-ri, Hwado-myeon, Ganghwa-gun, Incheon, Republic of Korea	2016-10-21	J. S. Park	1610105	GH5J						
Suaeda japonica Makino	Dongmak-ri, Hwado-myeon, Ganghwa-gun, Incheon, Republic of Korea	2016-10-21	J. S. Park	1610109	GH9J						

Suaeda japonica Makino	Dongmak-ri, Hwado-myeon, Ganghwa-gun, Incheon, Republic of Korea	2016-09-04	J. S. Park	1609002	GH11J	C	C	MT875070	MT873657	MT873753	
Suaeda japonica Makino	Dongmak-ri, Hwado-myeon, Ganghwa-gun, Incheon, Republic of Korea	2016-09-04	J. S. Park	1609004	GH13J						
Suaeda japonica Makino	Dongmak-ri, Hwado-myeon, Ganghwa-gun, Incheon, Republic of Korea	2016-09-04	J. S. Park	1609007	GH16J						
Suaeda japonica Makino	Dongmak-ri, Hwado-myeon, Ganghwa-gun, Incheon, Republic of Korea	2016-09-04	J. S. Park	1609010	GH19J	C	C	MT875074	MT873661	MT873757	
Suaeda japonica Makino	Dongmak-ri, Hwado-myeon, Ganghwa-gun, Incheon, Republic of Korea	2016-09-04	J. S. Park	1609013	GH22J						
Suaeda japonica Makino	Dongmak-ri, Hwado-myeon, Ganghwa-gun, Incheon, Republic of Korea	2016-09-04	J. S. Park	1609016	GH25J	C	C	MT875076	MT873663	MT873759	
Suaeda japonica Makino	Dongmak-ri, Hwado-myeon, Ganghwa-gun, Incheon, Republic of Korea	2016-09-04	J. S. Park	1609019	GH28J						
Suaeda japonica Makino	Dongmak-ri, Hwado-myeon, Ganghwa-gun, Incheon, Republic of Korea	2016-09-04	J. S. Park	-	GH31J	C	C	MT875077	MT873664	MT873760	
Suaeda japonica Makino	Doseong-ri estuary→Huansung-ri estuary, Jigok-myeon, Seosan-si, Chungcheongnam-do, Republic of Korea	2016-10-01	J. S. Park	1609331	SS1J	C	C	MT875124	MT873691	MT873787	
Suaeda japonica Makino	Doseong-ri estuary→Huansung-ri estuary, Jigok-myeon, Seosan-si, Chungcheongnam-do, Republic of Korea	2016-10-01	J. S. Park	1609333	SS3J						
Suaeda japonica Makino	Doseong-ri estuary→Huansung-ri estuary, Jigok-myeon, Seosan-si, Chungcheongnam-do, Republic of Korea	2016-10-01	J. S. Park	1609334	SS4J						
Suaeda japonica Makino	Doseong-ri estuary→Huansung-ri estuary, Jigok-myeon, Seosan-si, Chungcheongnam-do, Republic of Korea	2016-10-01	J. S. Park	1609335	SS5J	C	C	MT875125	MT873692	MT873788	
Suaeda japonica Makino	Doseong-ri estuary→Huansung-ri estuary, Jigok-myeon, Seosan-si, Chungcheongnam-do, Republic of Korea	2016-10-01	J. S. Park	1609339	SS9J						
Suaeda japonica Makino	Doseong-ri estuary→Huansung-ri estuary, Jigok-myeon, Seosan-si, Chungcheongnam-do, Republic of Korea	2016-10-01	J. S. Park	1609340	SS10J	C	C	MT875126	MT873693	MT873789	
Suaeda japonica Makino	Doseong-ri estuary→Huansung-ri estuary, Jigok-myeon, Seosan-si, Chungcheongnam-do, Republic of Korea	2016-10-01	J. S. Park	1609342	SS12J						
Suaeda japonica Makino	Doseong-ri estuary→Huansung-ri estuary, Jigok-myeon, Seosan-si, Chungcheongnam-do, Republic of Korea	2016-10-01	J. S. Park	1609344	SS14J	C	C	MT875127	MT873694	MT873790	
Suaeda japonica Makino	Doseong-ri estuary→Huansung-ri estuary, Jigok-myeon, Seosan-si, Chungcheongnam-do, Republic of Korea	2016-10-01	J. S. Park	1609346	SS16J						
Suaeda japonica Makino	Doseong-ri estuary→Huansung-ri estuary, Jigok-myeon, Seosan-si, Chungcheongnam-do, Republic of Korea	2016-10-01	J. S. Park	1609347	SS17J	C	C	MT875128	MT873695	MT873791	
Suaeda japonica Makino	Wa-tan stream riverside, Beopseong-po, Yeonggwang-gun, Jeollanam-do, Republic of Korea	2019-10-23	J. S. Park	1910381	YGY1J	C	C	MT875129	MT873696	MT873792	
Suaeda japonica Makino	Wa-tan stream riverside, Beopseong-po, Yeonggwang-gun, Jeollanam-do, Republic of Korea	2019-10-23	J. S. Park	1910383	YGY3J						
Suaeda japonica Makino	Wa-tan stream riverside, Beopseong-po, Yeonggwang-gun, Jeollanam-do, Republic of Korea	2019-10-23	J. S. Park	1910385	YGY5J	C	C	MT875130	MT873697	MT873793	
Suaeda japonica Makino	Wa-tan stream riverside, Beopseong-po, Yeonggwang-gun, Jeollanam-do, Republic of Korea	2019-10-23	J. S. Park	1910387	YGY7J						
Suaeda japonica Makino	Wa-tan stream riverside, Beopseong-po, Yeonggwang-gun, Jeollanam-do, Republic of Korea	2019-10-23	J. S. Park	1910389	YGY9J	C	C	MT875131	MT873698	MT873794	
Suaeda japonica Makino	Wa-tan stream riverside, Beopseong-po, Yeonggwang-gun, Jeollanam-do, Republic of Korea	2019-10-23	J. S. Park	1910391	YGY11J						
Suaeda japonica Makino	Wa-tan stream riverside, Beopseong-po, Yeonggwang-gun, Jeollanam-do, Republic of Korea	2019-10-23	J. S. Park	1910393	YGY13J						
Suaeda japonica Makino	Wa-tan stream riverside, Beopseong-po, Yeonggwang-gun, Jeollanam-do, Republic of Korea	2019-10-23	J. S. Park	1910395	YGY15J	C	C	MT875132	MT873699	MT873795	
Suaeda japonica Makino	Wa-tan stream riverside, Beopseong-po, Yeonggwang-gun, Jeollanam-do, Republic of Korea	2019-10-23	J. S. Park	1910397	YGY17J						
Suaeda japonica Makino	Wa-tan stream riverside, Beopseong-po, Yeonggwang-gun, Jeollanam-do, Republic of Korea	2019-10-23	J. S. Park	1910398	YGY18J						
Suaeda japonica Makino	Wa-tan stream riverside, Beopseong-po, Yeonggwang-gun, Jeollanam-do, Republic of Korea	2019-10-23	J. S. Park	1910400	YGY20J	C	C	MT875133	MT873700	MT873796	
Suaeda japonica Makino	Near In-an bridge, Anpung-dong, Suncheon-si, Jeollanam-do, Republic of Korea	2016-10-14	J. S. Park	1610041	SCH1J	C	C	MT875114	MT873681	MT873777	
Suaeda japonica Makino	Abandoned fish farm, Haksan-ri, Byeollyang-myeon, Suncheon-si, Jeollanam-do, Republic of Korea	2016-10-14	J. S. Park	1610045	SCH5J						
Suaeda japonica Makino	Abandoned fish farm, Haksan-ri, Byeollyang-myeon, Suncheon-si, Jeollanam-do, Republic of Korea	2016-10-14	J. S. Park	1610051	SCH11J	C	C	MT875115	MT873682	MT873778	
Suaeda japonica Makino	Abandoned fish farm, Haksan-ri, Byeollyang-myeon, Suncheon-si, Jeollanam-do, Republic of Korea	2016-10-14	J. S. Park	1610062	SCH22J						
Suaeda japonica Makino	Mudflat, Nongju-ri, Haeryong-myeon, Suncheon-si, Jeollanam-do, Republic of Korea	2019-10-23	J. S. Park	1910361	SCH31J						
Suaeda japonica Makino	Mudflat, Nongju-ri, Haeryong-myeon, Suncheon-si, Jeollanam-do, Republic of Korea	2019-10-23	J. S. Park	1910364	SCH34J	C	C	MT875116	MT873683	MT873779	
Suaeda japonica Makino	Mudflat, Nongju-ri, Haeryong-myeon, Suncheon-si, Jeollanam-do, Republic of Korea	2019-10-23	J. S. Park	1910365	SCH35J						
Suaeda japonica Makino	Mudflat, Nongju-ri, Haeryong-myeon, Suncheon-si, Jeollanam-do, Republic of Korea	2019-10-23	J. S. Park	1910367	SCH37J	C	C	MT875117	MT873684	MT873780	
Suaeda japonica Makino	Mudflat, Nongju-ri, Haeryong-myeon, Suncheon-si, Jeollanam-do, Republic of Korea	2019-10-23	J. S. Park	1910370	SCH40J						
Suaeda japonica Makino	Mudflat, Nongju-ri, Haeryong-myeon, Suncheon-si, Jeollanam-do, Republic of Korea	2019-10-23	J. S. Park	1910373	SCH43J						
Suaeda japonica Makino	Mudflat, Nongju-ri, Haeryong-myeon, Suncheon-si, Jeollanam-do, Republic of Korea	2019-10-23	J. S. Park	1910375	SCH45J	C	C	MT875118	MT873685	MT873781	
Suaeda japonica Makino	Mutsugorou Park, Ogi-shi, Saga Prefecture, Japan	2017-09-24	J. S. Park & J. W. Park	1709301	OG5J	D	C	MT875102	MT873639	MT873735	
Suaeda japonica Makino	Higatayaka Park, Saga-shi, Saga Prefecture, Japan	2017-09-24	J. S. Park & J. W. Park	1709302	OG6J						
Suaeda japonica Makino	Mutsugorou Park, Ogi-shi, Saga Prefecture, Japan	2017-09-24	J. S. Park & J. W. Park	1709401	OG1J	D	C	MT875098	MT873635	MT873731	
Suaeda japonica Makino	Mutsugorou Park, Ogi-shi, Saga Prefecture, Japan	2017-09-24	J. S. Park & J. W. Park	1709402	OG2J	D	C	MT875099	MT873636	MT873732	
Suaeda japonica Makino	Mutsugorou Park, Ogi-shi, Saga Prefecture, Japan	2017-09-24	J. S. Park & J. W. Park	1709403	OG3J	D	C	MT875100	MT873637	MT873733	
Suaeda japonica Makino	Mutsugorou Park, Ogi-shi, Saga Prefecture, Japan	2017-09-24	J. S. Park & J. W. Park	1709404	OG4J	D	C	MT875101	MT873638	MT873734	
Suaeda maritima (L.) Dumort.	Around farmland, Sagi-ri, Hwado-myeon, Ganghwa-gun, Incheon, Republic of Korea	2019-10-16	J. S. Park	1910321	GH1M	C	D	MT875065	MT873652	MT873748	Heteroptera lineage
Suaeda maritima (L.) Dumort.	Around farmland, Sagi-ri, Hwado-myeon, Ganghwa-gun, Incheon, Republic of Korea	2019-10-16	J. S. Park	1910322	GH2M						Heteroptera lineage

Suaeda maritima (L.) Dumort.	Around farmland, Sagi-ri, Hwado-myeon, Ganghwa-gun, Incheon, Republic of Korea	2019-10-16	J. S. Park	1910324	GH4M	C	D	MT875067	MT873654	MT873750	Heteroptera lineage
Suaeda maritima (L.) Dumort.	Around farmland, Sagi-ri, Hwado-myeon, Ganghwa-gun, Incheon, Republic of Korea	2019-10-16	J. S. Park	1910325	GH5M						Heteroptera lineage
Suaeda maritima (L.) Dumort.	Around farmland, Sagi-ri, Hwado-myeon, Ganghwa-gun, Incheon, Republic of Korea	2019-10-16	J. S. Park	1910327	GH7M						Heteroptera lineage
Suaeda maritima (L.) Dumort.	Around farmland, Sagi-ri, Hwado-myeon, Ganghwa-gun, Incheon, Republic of Korea	2019-10-16	J. S. Park	1910328	GH8M	C	C	MT875069	MT873656	MT873752	Heteroptera lineage
Suaeda maritima (L.) Dumort.	Around farmland, Sagi-ri, Hwado-myeon, Ganghwa-gun, Incheon, Republic of Korea	2019-10-16	J. S. Park	1910330	GH10M						Heteroptera lineage
Suaeda maritima (L.) Dumort.	Around farmland, Sagi-ri, Hwado-myeon, Ganghwa-gun, Incheon, Republic of Korea	2019-10-16	J. S. Park	1910331	GH11M	C	D	MT875071	MT873658	MT873754	Heteroptera lineage
Suaeda maritima (L.) Dumort.	Around farmland, Sagi-ri, Hwado-myeon, Ganghwa-gun, Incheon, Republic of Korea	2019-10-16	J. S. Park	1910333	GH13M						Heteroptera lineage
Suaeda maritima (L.) Dumort.	Around farmland, Sagi-ri, Hwado-myeon, Ganghwa-gun, Incheon, Republic of Korea	2019-10-16	J. S. Park	1910334	GH14M						Heteroptera lineage
Suaeda maritima (L.) Dumort.	Around farmland, Sagi-ri, Hwado-myeon, Ganghwa-gun, Incheon, Republic of Korea	2019-10-16	J. S. Park	1910336	GH16M	C	C	MT875073	MT873660	MT873756	Heteroptera lineage
Suaeda maritima (L.) Dumort.	Around fish farm, Shin-chang stream, Boan-myeon, Buan-gun, Jeollabuk-do, Republic of Korea	2016-10-23	J. S. Park	1610161	BU1M	C	F	MT875053	MT873645	MT873741	Heteroptera lineage
Suaeda maritima (L.) Dumort.	Around fish farm, Shin-chang stream, Boan-myeon, Buan-gun, Jeollabuk-do, Republic of Korea	2016-10-23	J. S. Park	1610164	BU4M						Heteroptera lineage
Suaeda maritima (L.) Dumort.	Around fish farm, Shin-chang stream, Boan-myeon, Buan-gun, Jeollabuk-do, Republic of Korea	2016-10-23	J. S. Park	1610166	BU6M	C	C	MT875054	MT873646	MT873742	Heteroptera lineage
Suaeda maritima (L.) Dumort.	Around fish farm, Shin-chang stream, Boan-myeon, Buan-gun, Jeollabuk-do, Republic of Korea	2016-10-23	J. S. Park	1610168	BU8M						Heteroptera lineage
Suaeda maritima (L.) Dumort.	Around fish farm, Shin-chang stream, Boan-myeon, Buan-gun, Jeollabuk-do, Republic of Korea	2016-10-23	J. S. Park	1610170	BU10M	C	F	MT875055	MT873647	MT873743	Heteroptera lineage
Suaeda maritima (L.) Dumort.	Around fish farm, Shin-chang stream, Boan-myeon, Buan-gun, Jeollabuk-do, Republic of Korea	2016-10-23	J. S. Park	1610176	BU16M						Heteroptera lineage
Suaeda maritima (L.) Dumort.	Around fish farm, Shin-chang stream, Boan-myeon, Buan-gun, Jeollabuk-do, Republic of Korea	2016-10-23	J. S. Park	1610179	BU19M	C	F	MT875056	MT873648	MT873744	Heteroptera lineage
Suaeda maritima (L.) Dumort.	Around fish farm, Shin-chang stream, Boan-myeon, Buan-gun, Jeollabuk-do, Republic of Korea	2016-10-23	J. S. Park	1610182	BU22M						Heteroptera lineage
Suaeda maritima (L.) Dumort.	Around fish farm, Shin-chang stream, Boan-myeon, Buan-gun, Jeollabuk-do, Republic of Korea	2016-10-23	J. S. Park	1610185	BU25M						Heteroptera lineage
Suaeda maritima (L.) Dumort.	Around fish farm, Shin-chang stream, Boan-myeon, Buan-gun, Jeollabuk-do, Republic of Korea	2016-10-23	J. S. Park	1610188	BU28M	C	F	MT875057	MT873649	MT873745	Heteroptera lineage
Suaeda maritima (L.) Dumort.	Fish farm, Janggam-ri, Aphae-eup, Sinan-gun, Jeollanam-do, Republic of Korea	2019-10-19	J. S. Park	1910341	SAN1M	C	F	MT875108	MT873675	MT873771	Heteroptera lineage
Suaeda maritima (L.) Dumort.	Fish farm, Janggam-ri, Aphae-eup, Sinan-gun, Jeollanam-do, Republic of Korea	2019-10-19	J. S. Park	1910342	SAN2M						Heteroptera lineage
Suaeda maritima (L.) Dumort.	Fish farm, Janggam-ri, Aphae-eup, Sinan-gun, Jeollanam-do, Republic of Korea	2019-10-19	J. S. Park	1910343	SAN3M	C	C	MT875109	MT873676	MT873772	Heteroptera lineage
Suaeda maritima (L.) Dumort.	Fish farm, Janggam-ri, Aphae-eup, Sinan-gun, Jeollanam-do, Republic of Korea	2019-10-19	J. S. Park	1910345	SAN5M						Heteroptera lineage
Suaeda maritima (L.) Dumort.	Fish farm, Janggam-ri, Aphae-eup, Sinan-gun, Jeollanam-do, Republic of Korea	2019-10-19	J. S. Park	1910346	SAN6M	C	F	MT875110	MT873677	MT873773	Heteroptera lineage
Suaeda maritima (L.) Dumort.	Fish farm, Janggam-ri, Aphae-eup, Sinan-gun, Jeollanam-do, Republic of Korea	2019-10-19	J. S. Park	1910347	SAN7M						Heteroptera lineage
Suaeda maritima (L.) Dumort.	Fish farm, Janggam-ri, Aphae-eup, Sinan-gun, Jeollanam-do, Republic of Korea	2019-10-19	J. S. Park	1910348	SAN8M						Heteroptera lineage
Suaeda maritima (L.) Dumort.	Fish farm, Janggam-ri, Aphae-eup, Sinan-gun, Jeollanam-do, Republic of Korea	2019-10-19	J. S. Park	1910349	SAN9M	C	F	MT875111	MT873678	MT873774	Heteroptera lineage
Suaeda maritima (L.) Dumort.	Fish farm, Janggam-ri, Aphae-eup, Sinan-gun, Jeollanam-do, Republic of Korea	2019-10-19	J. S. Park	1910350	SAN10M						Heteroptera lineage
Suaeda maritima (L.) Dumort.	Fish farm, Janggam-ri, Aphae-eup, Sinan-gun, Jeollanam-do, Republic of Korea	2019-10-19	J. S. Park	1910351	SAN11M						Heteroptera lineage
Suaeda maritima (L.) Dumort.	Fish farm, Janggam-ri, Aphae-eup, Sinan-gun, Jeollanam-do, Republic of Korea	2019-10-19	J. S. Park	1910352	SAN12M	C	C	MT875112	MT873679	MT873775	Heteroptera lineage
Suaeda heteroptera Kitag.	Around Xian port, Qidong Shi, Nantong Shi, Jiangsu Sheng, China	2019-10-10	J. S. Park & Y. J. Jang	1910202	QDO2M	E	F	MT875103	MT873610	MT873706	Heteroptera lineage
Suaeda heteroptera Kitag.	Around Xian port, Qidong Shi, Nantong Shi, Jiangsu Sheng, China	2019-10-10	J. S. Park & Y. J. Jang	1910203	QDO3M						Heteroptera lineage
Suaeda heteroptera Kitag.	Around Xian port, Qidong Shi, Nantong Shi, Jiangsu Sheng, China	2019-10-10	J. S. Park & Y. J. Jang	-	QDO6M	E	E	MT875104	MT873611	MT873707	Heteroptera lineage
Suaeda heteroptera Kitag.	Around Xian port, Qidong Shi, Nantong Shi, Jiangsu Sheng, China	2019-10-10	J. S. Park & Y. J. Jang	-	QDO7M						Heteroptera lineage
Suaeda heteroptera Kitag.	Around Xian port, Qidong Shi, Nantong Shi, Jiangsu Sheng, China	2019-10-10	J. S. Park & Y. J. Jang	-	QDO9M						Heteroptera lineage
Suaeda heteroptera Kitag.	Around Xian port, Qidong Shi, Nantong Shi, Jiangsu Sheng, China	2019-10-10	J. S. Park & Y. J. Jang	-	QDO11M	E	F	MT875105	MT873612	MT873708	Heteroptera lineage
Suaeda heteroptera Kitag.	Around road near Farm Sizu, Qidong Shi, Nantong Shi, Jiangsu Sheng, China	2019-10-11	J. S. Park & Y. J. Jang	-	QDO13M						Heteroptera lineage
Suaeda heteroptera Kitag.	Around road near Farm Sizu, Qidong Shi, Nantong Shi, Jiangsu Sheng, China	2019-10-11	J. S. Park & Y. J. Jang	1910216	QDO16M	E	F	MT875106	MT873613	MT873709	Heteroptera lineage
Suaeda heteroptera Kitag.	Around road near Farm Sizu, Qidong Shi, Nantong Shi, Jiangsu Sheng, China	2019-10-11	J. S. Park & Y. J. Jang	-	QDO18M						Heteroptera lineage
Suaeda heteroptera Kitag.	Around road near Farm Sizu, Qidong Shi, Nantong Shi, Jiangsu Sheng, China	2019-10-11	J. S. Park & Y. J. Jang	1910220	QDO20M	C	F	MT875107	MT873614	MT873710	Heteroptera lineage
Suaeda heteroptera Kitag.	Guanyun, Lianyungang Shi, Jiangsu Sheng, China	2019-10-12	J. S. Park & Y. J. Jang	1910271	GYU1M	C	E	MT875078	MT873605	MT873701	Heteroptera lineage
Suaeda heteroptera Kitag.	Guanyun, Lianyungang Shi, Jiangsu Sheng, China	2019-10-12	J. S. Park & Y. J. Jang	-	GYU2M						Heteroptera lineage
Suaeda heteroptera Kitag.	Guanyun, Lianyungang Shi, Jiangsu Sheng, China	2019-10-12	J. S. Park & Y. J. Jang	-	GYU3M						Heteroptera lineage
Suaeda heteroptera Kitag.	Guanyun, Lianyungang Shi, Jiangsu Sheng, China	2019-10-12	J. S. Park & Y. J. Jang	-	GYU5M	C	C	MT875079	MT873606	MT873702	Heteroptera lineage
Suaeda heteroptera Kitag.	Guanyun, Lianyungang Shi, Jiangsu Sheng, China	2019-10-12	J. S. Park & Y. J. Jang	-	GYU6M						Heteroptera lineage
Suaeda heteroptera Kitag.	Guanyun, Lianyungang Shi, Jiangsu Sheng, China	2019-10-12	J. S. Park & Y. J. Jang	1910278	GYU8M	C	E	MT875080	MT873607	MT873703	Heteroptera lineage
Suaeda heteroptera Kitag.	Guanyun, Lianyungang Shi, Jiangsu Sheng, China	2019-10-12	J. S. Park & Y. J. Jang	-	GYU9M						Heteroptera lineage
Suaeda heteroptera Kitag.	Guanyun, Lianyungang Shi, Jiangsu Sheng, China	2019-10-12	J. S. Park & Y. J. Jang	-	GYU11M	E	F	MT875081	MT873608	MT873704	Heteroptera lineage

Suaeda heteroptera Kitag.	Guanyun, Lianyungang Shi, Jiangsu Sheng, China	2019-10-12	J. S. Park & Y. J. Jang	-	GYU12M						Heteroptera lineage
Suaeda heteroptera Kitag.	Guanyun, Lianyungang Shi, Jiangsu Sheng, China	2019-10-12	J. S. Park & Y. J. Jang	-	GYU13M						Heteroptera lineage
Suaeda heteroptera Kitag.	Guanyun, Lianyungang Shi, Jiangsu Sheng, China	2019-10-12	J. S. Park & Y. J. Jang	-	GYU14M	E	E	MT875082	MT873609	MT873705	Heteroptera lineage
Suaeda glauca (Bunge) Bunge	Janghang pine forest→Baeksa village along the beach, Seocheon-gun, Chungcheongnam-do, Republic of Korea	2016-09-29	J. S. Park & D. P. Jin	1609126	SC1G			MT875113	MT873680	MT873776	

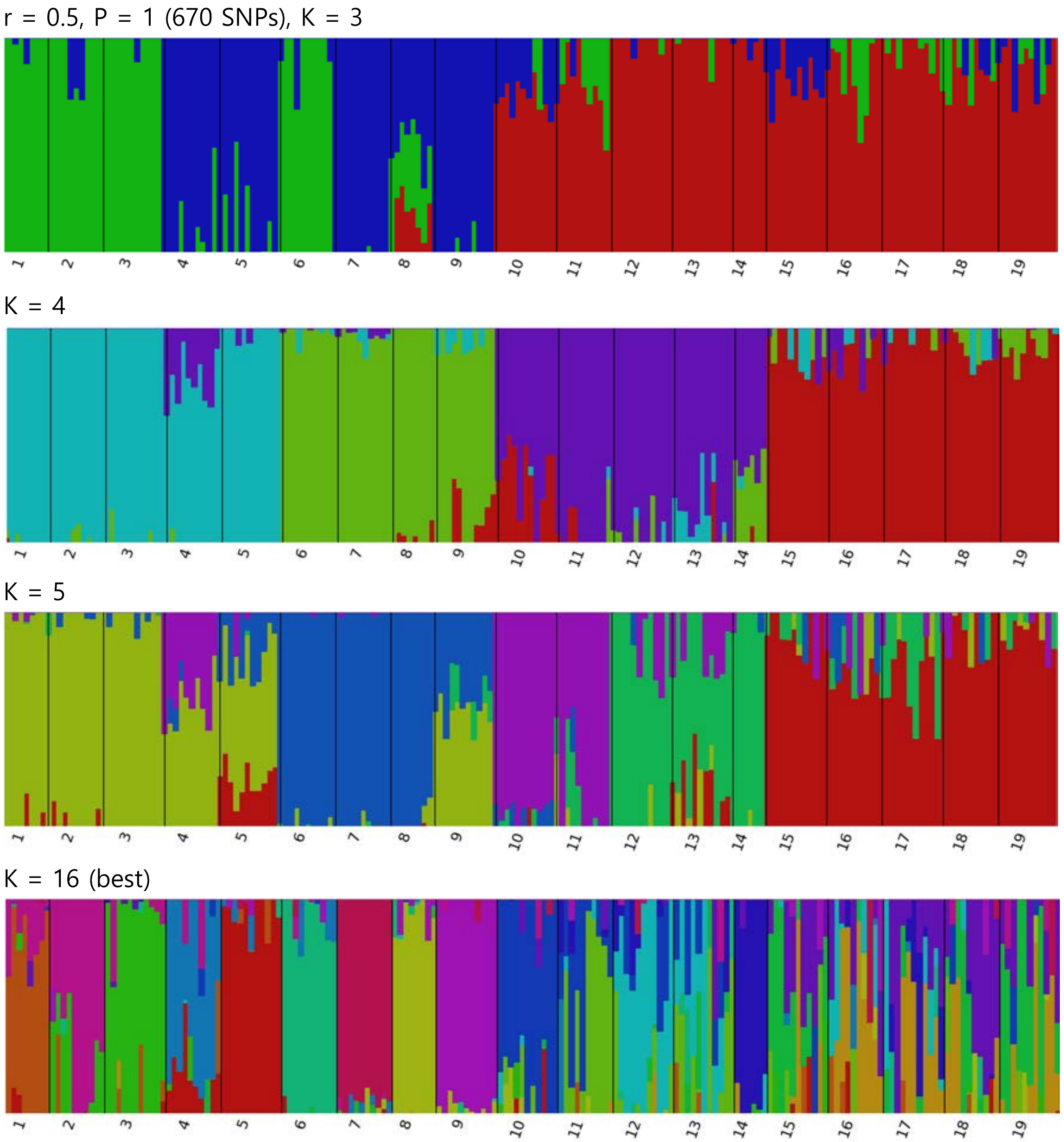

r = 0.5, P = 1 (670 SNPs), K = 3
1 2 3 4 5 6 7 8 9 10 11 12 13 14 15 16 17 18 19
K = 4
1 2 3 4 5 6 7 8 9 10 11 12 13 14 15 16 17 18 19
K = 5
1 2 3 4 5 6 7 8 9 10 11 12 13 14 15 16 17 18 19
K = 16 (best)
1 2 3 4 5 6 7 8 9 10 11 12 13 14 15 16 17 18 19

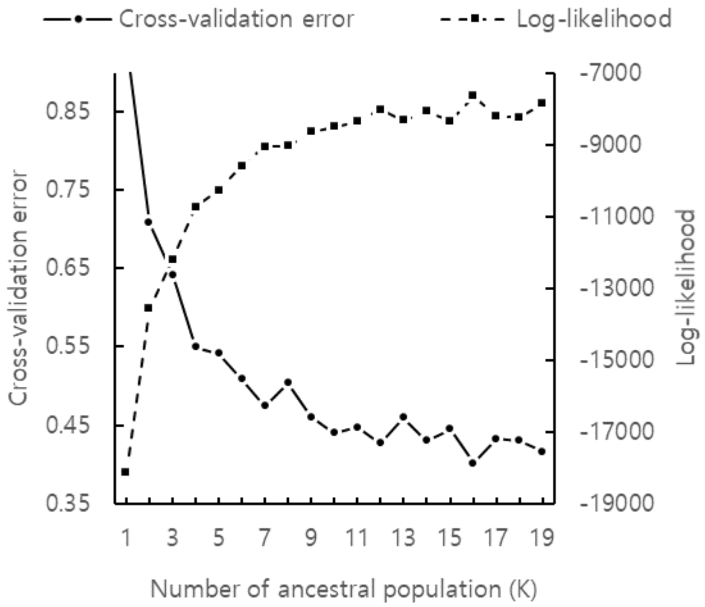

Cross-validation error
Log-likelihood
0.85
0.75
0.65
0.55
0.45
0.35
-7000
-9000
-11000
-13000
-15000
-17000
-19000
1 3 5 7 9 11 13 15 17 19
Number of ancestral population (K)

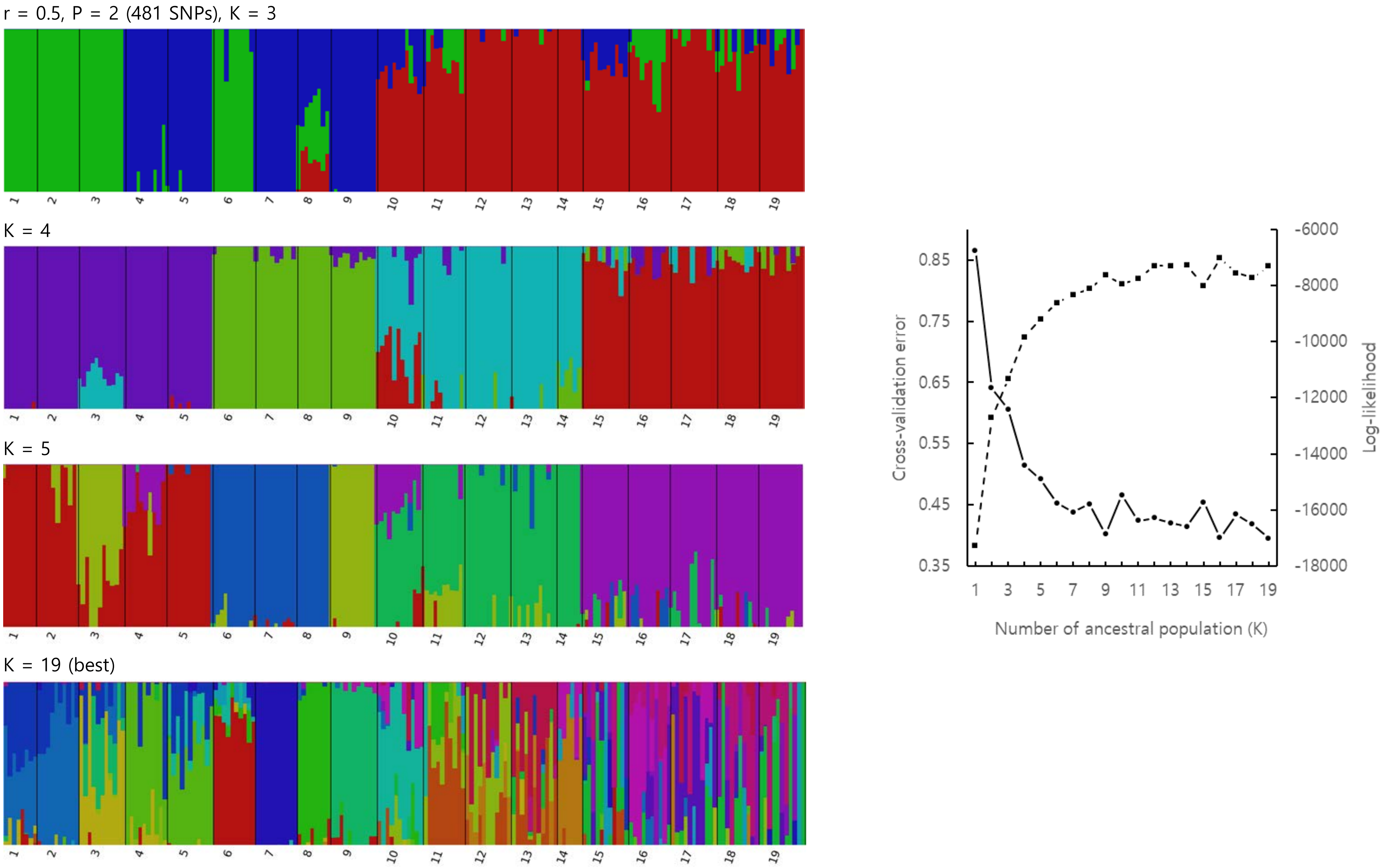
r = 0.5, P = 2 (481 SNPs), K = 3
K = 4
K = 5
K = 19 (best)
1 2 3 4 5 6 7 8 9 10 11 12 13 14 15 16 17 18 19
Cross-validation error
0.85
0.75
0.65
0.55
0.45
0.35
Log-likelihood
-6000
-8000
-10000
-12000
-14000
-16000
-18000
1 3 5 7 9 11 13 15 17 19
Number of ancestral population (K)

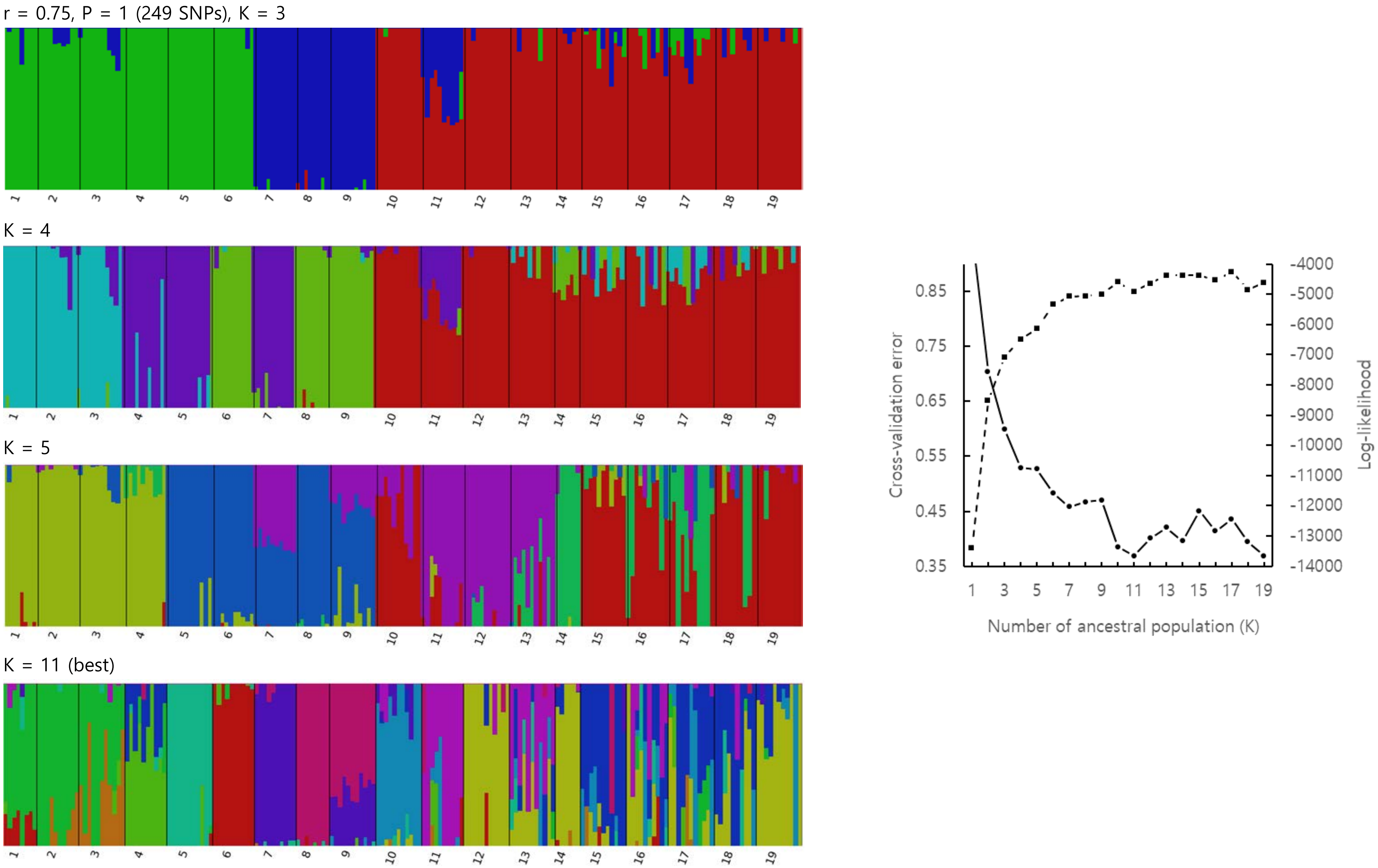

r = 0.75, P = 1 (249 SNPs), K = 3
1 2 3 4 5 6 7 8 9 10 11 12 13 14 15 16 17 18 19
K = 4
1 2 3 4 5 6 7 8 9 10 11 12 13 14 15 16 17 18 19
K = 5
1 2 3 4 5 6 7 8 9 10 11 12 13 14 15 16 17 18 19
K = 11 (best)
1 2 3 4 5 6 7 8 9 10 11 12 13 14 15 16 17 18 19
Cross-validation error
0.85
0.75
0.65
0.55
0.45
0.35
Log-likelihood
-4000
-5000
-6000
-7000
-8000
-9000
-10000
-11000
-12000
-13000
-14000
1 3 5 7 9 11 13 15 17 19
Number of ancestral population (K)

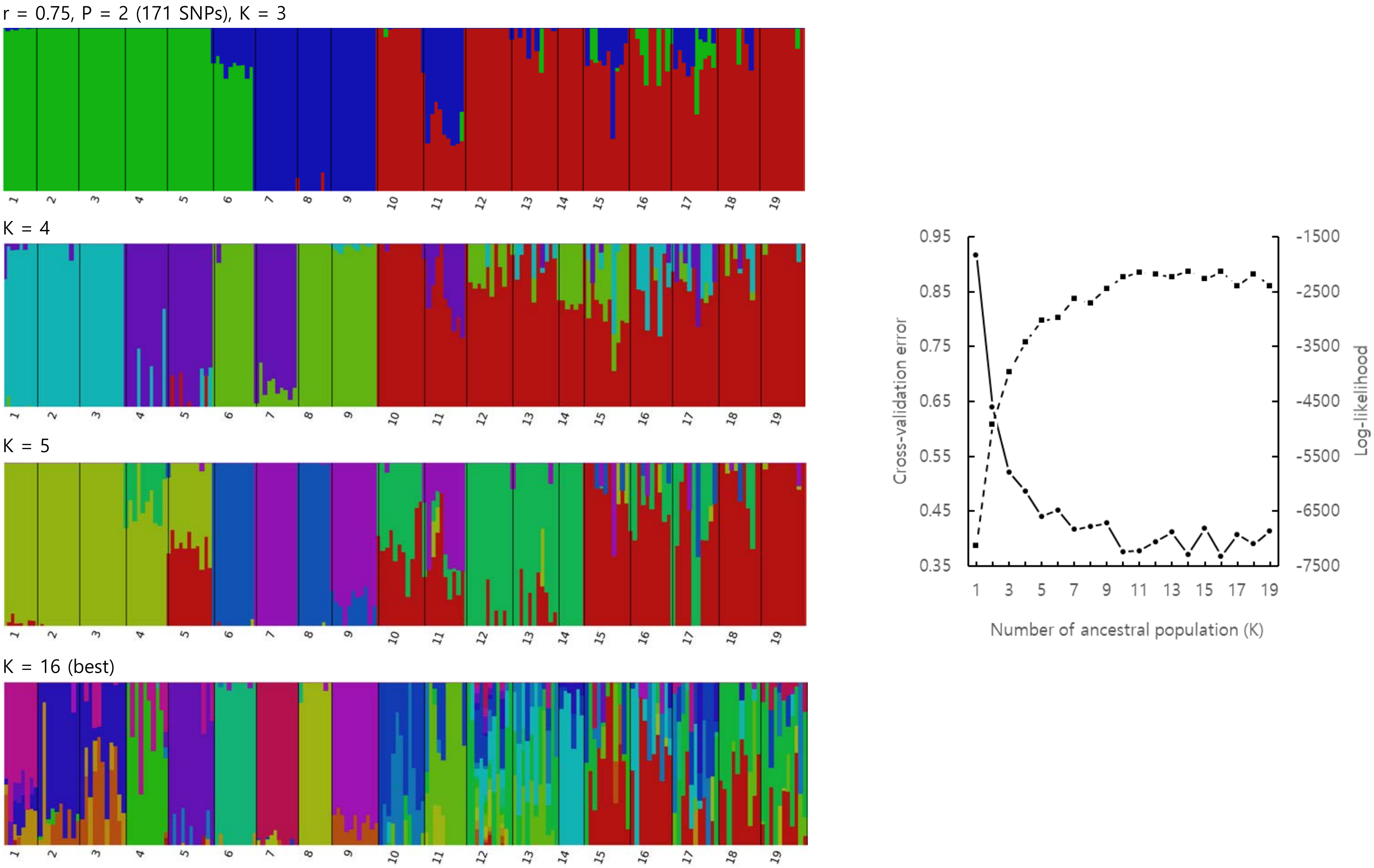

Figure S1. Admixture analysis of first SNP dataset with other parameters.

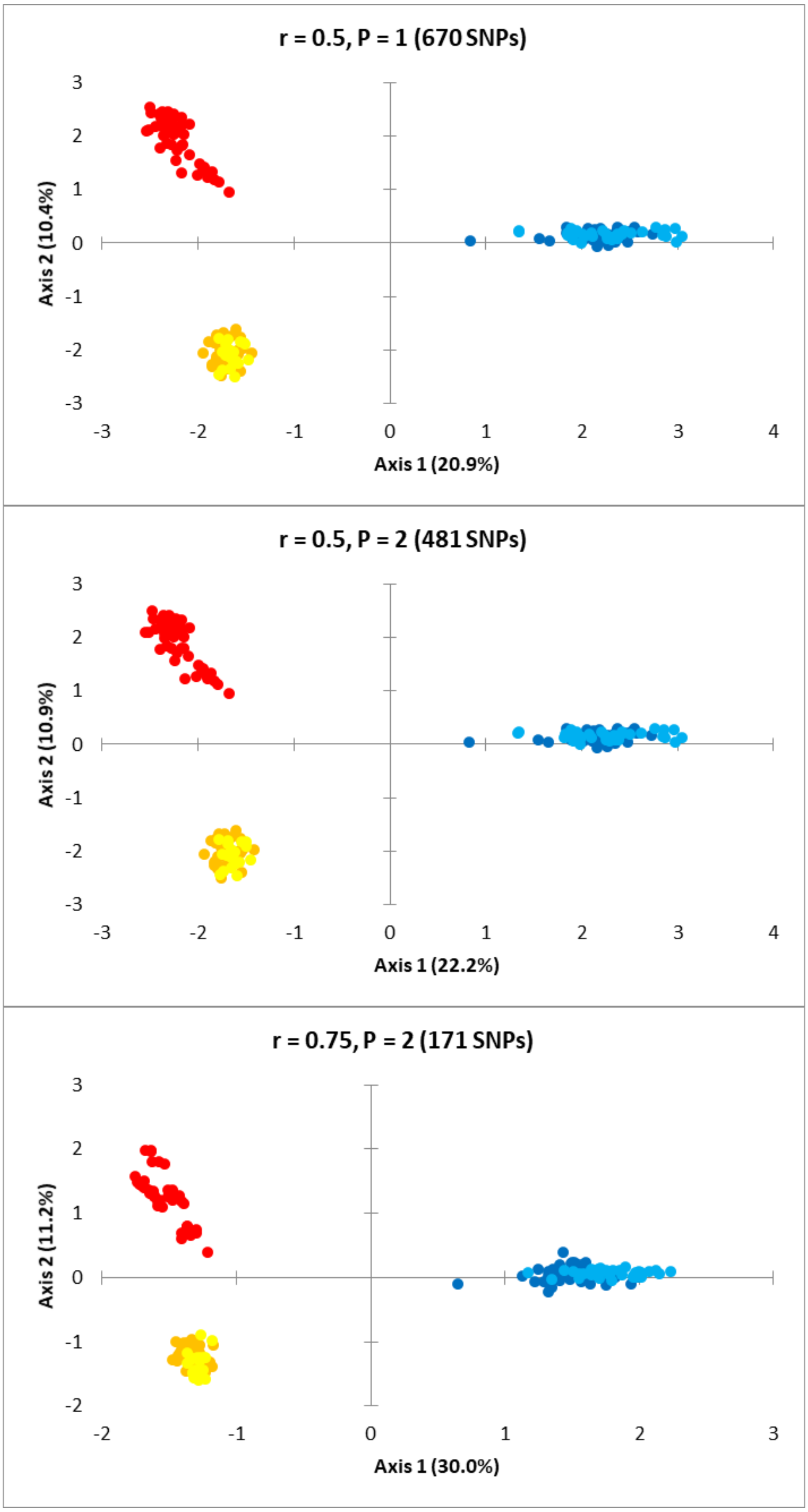

Figure S2. PCoA analysis with other parameters.

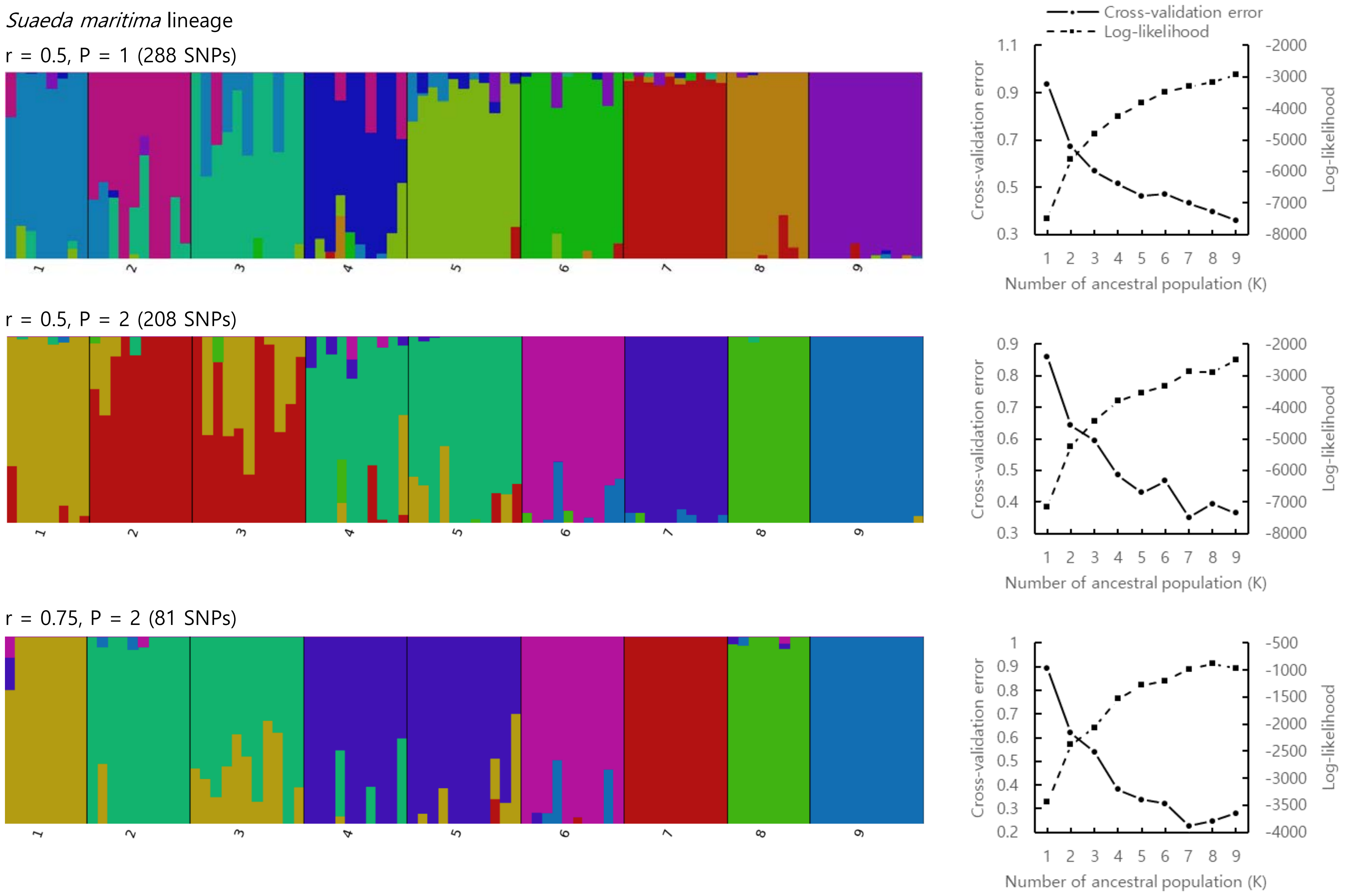

Suaeda maritima lineage
r = 0.5, P = 1 (288 SNPs)
r = 0.5, P = 2 (208 SNPs)
r = 0.75, P = 2 (81 SNPs)
1 2 3 4 5 6 7 8 9
Cross-validation error
Log-likelihood
Number of ancestral population (K)

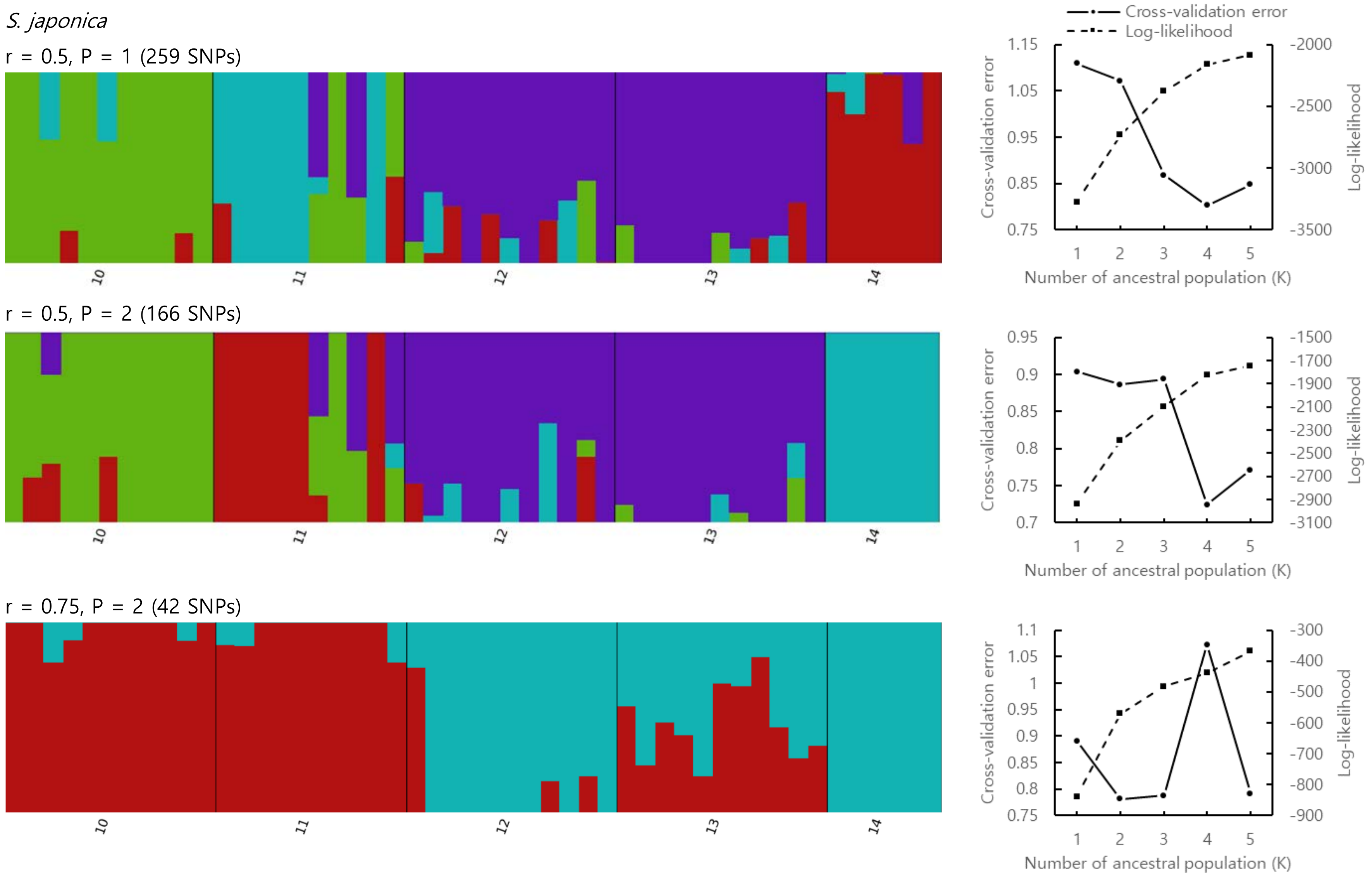

Figure S3. Admixture analysis from second datasets of *Suaeda maritima* lineage and *S. japonica* with other parameters.

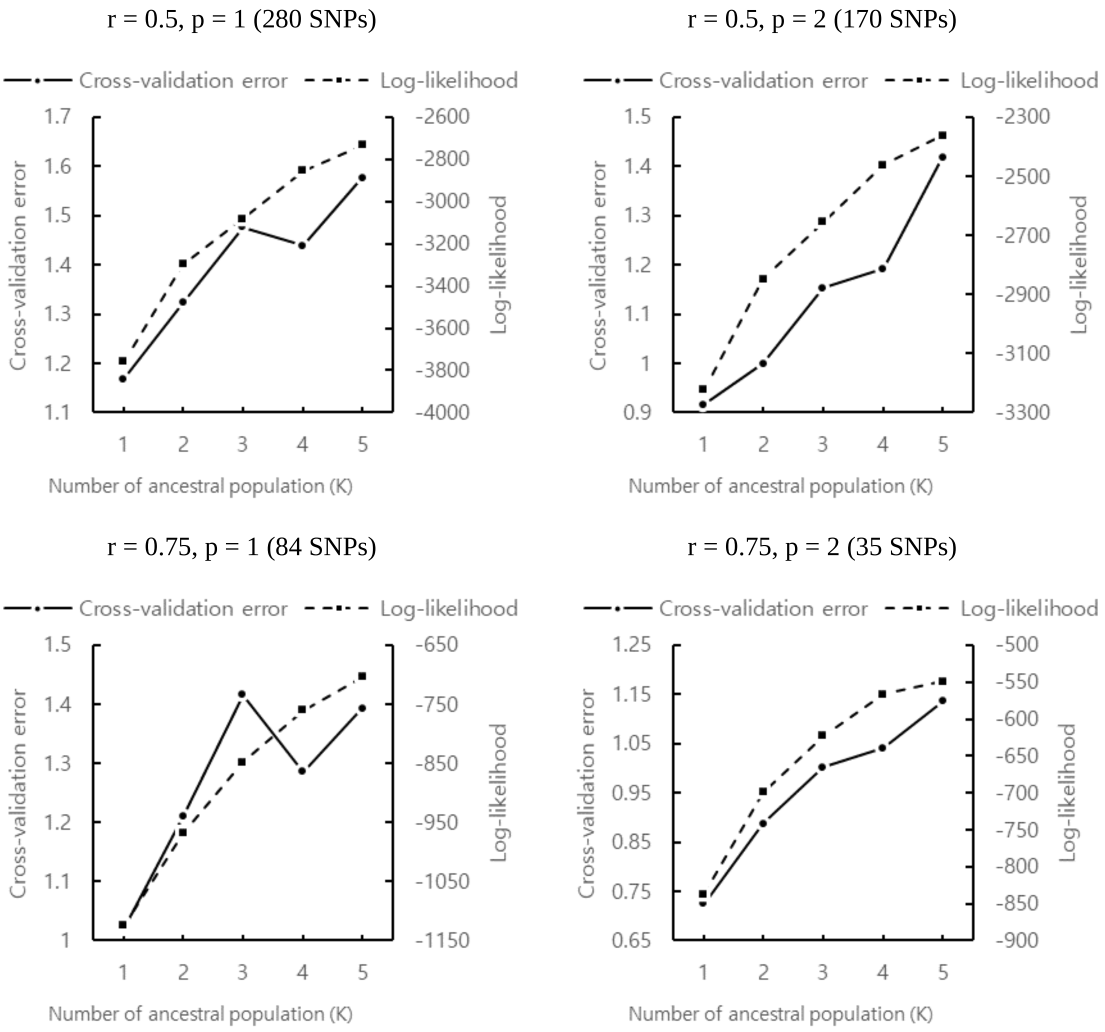

Figure S4. Cross-validation and log-likelihood in Admixture analysis from second dataset of *Suaeda heteroptera* lineage

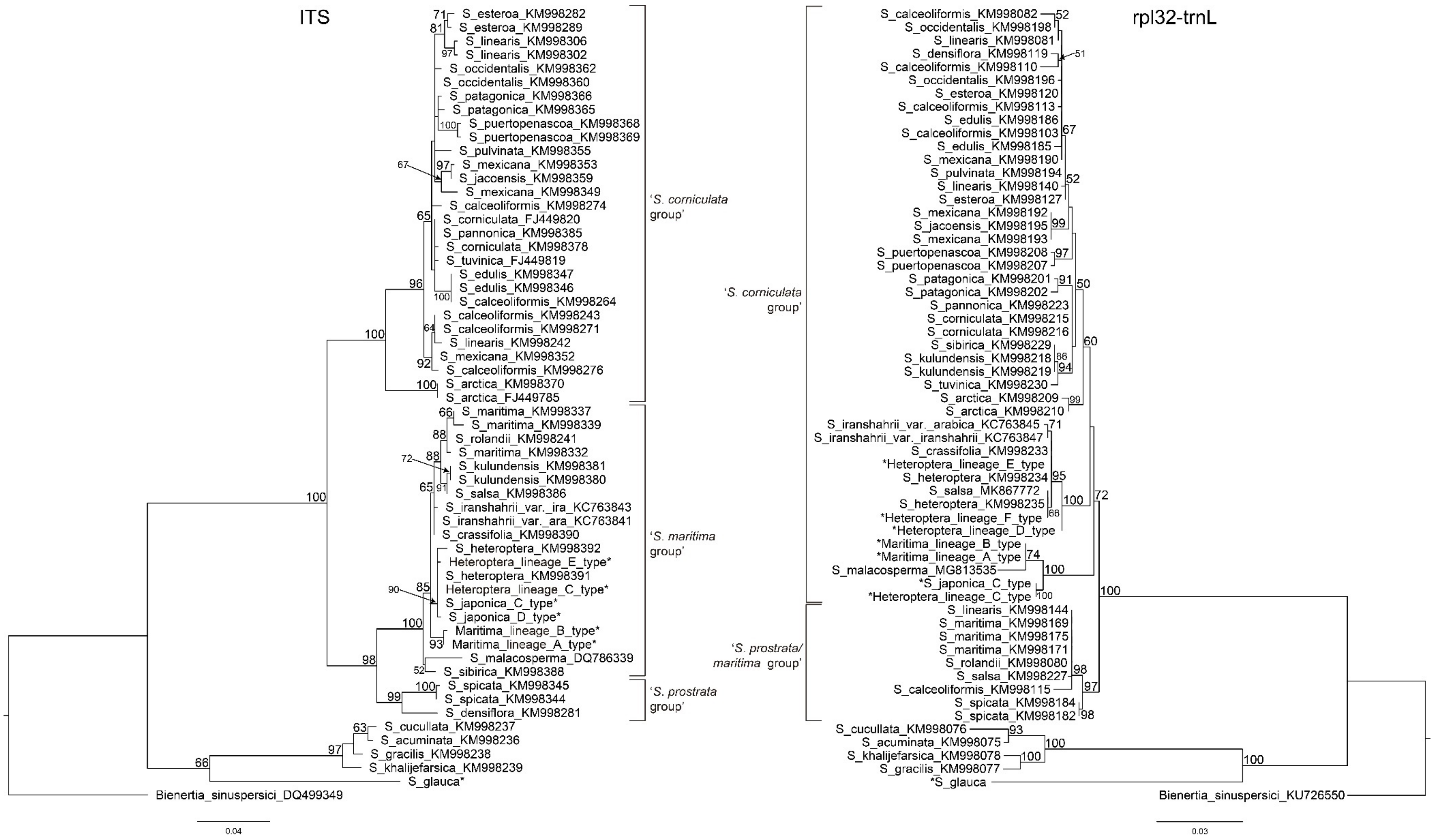

Figure S5. Phylogenetic trees of cpDNA (*rpl32-trnL*) and nDNA (ITS). Each genotype in both trees is corresponding with that in Fig. 1. Each group is referenced in Brandt et al.[5]
